# Spatiotemporal patterns of gene expression changes in the mouse dentate gyrus following entorhinal denervation

**DOI:** 10.3389/fnmol.2026.1758390

**Published:** 2026-05-19

**Authors:** Jessica Schlaudraff, Domenico Del Turco, Jana Key, Thomas Deller, Georg Auburger

**Affiliations:** 1 Institute of Clinical Neuroanatomy, Goethe University Frankfurt, Frankfurt am Main, Germany; 2 Clinic of Neurology, Exp. Neurology, Goethe University Frankfurt, University Hospital, Frankfurt am Main, Germany

**Keywords:** entorhinal cortex lesion, gene set enrichment analysis, hippocampus, microarray analysis, protein-protein-interaction

## Abstract

**Introduction:**

The central nervous system responds to acute injury with plastic remodeling of its network. However, the temporal and structural dynamics of this response in the denervated dentate gyrus remain poorly understood. Therefore, we examined the transcriptional programs activated after perforant path transection, focusing on the outer molecular layer (OML) and the granule cell layer (GCL).

**Methods:**

Perforant path transections were performed, and tissue from the denervated OML and GCL was selectively isolated using laser microdissection at 1, 3, 7, 14, and 28 days post-lesion. Whole-genome expression analysis was performed to identify and characterize global transcriptome changes across time and layers.

**Results:**

Denervation induced reactive astrogliosis and microgliosis primarily in the OML. Overall enrichment statistics from two complementary approaches highlighted early downregulations of neuroactive ligands, later downregulation of (mainly glutamatergic) neurotransmission factors, and late downregulations of respiratory factors, alongside early upregulations of debris degradation factors, subsequent upregulation of glial and inflammatory factors, and late upregulation of ribosomal translation. Entorhinal afferent loss was reflected by reductions of the axon repellent *Sema3e* (to 60% in OML), with sustained depletions of the reelin repressor *Adamts3* (14% in OML, 33% in GCL) and its presumptive effector *Ptpn3* (30% in OML), as well as delayed induction of the SEMA3E/reelin coreceptor *Nrp1* (3-fold in OML, 7-fold in GCL). Secondary reductions in the GCL occurred for the CREB repressor *Rgs13*, *Ntng1*, and *Epha5*–*Epha6*–*Epha7*. Deficits were found in the OML for glutamatergic signaling factors (*Neto1*, *Cnih2*, *Dlg2*, *Gria1*, *Grm7*–*Grm8*–*Grm3*, *Homer2, Trim32*), in the GCL for GABAergic and glycinergic receptors (*Gabra2*–*Gabra1*, *Gabrb2*, *Glra2*), and overall for neuronal differentiation markers (*Calb1*, *Pvalb*, *Gad1*, *Lrrc7, Ano3*, *Bdnf*, *Egfr*, *Igf2r*, *Nrep*, *Basp1*, *Scn1a*) and synaptic adhesion mediators (*Lingo2*, *Lrrtm1*–*Lrrtm3*, *Adam11*–*Adam23*, *Cadm2*, *Pcdhb16*, *Flrt3*). Presumed compensatory upregulation efforts included postsynaptic and dendritic membrane remodeling by vesicle and actin regulators (*Lyn*, *Gabarap*, *Cyfip1*, *Arf4*, *Arl4c*, *Rab8b*, *Cdk5rap2*, *Tmsb4x*) in the OML, versus neurotrophic coreceptors *Sorcs3*–*Sorcs2*, axonal *Smn1*, and the neural progenitor modulator *Nup210l* in the GCL.

**Discussion:**

These findings identify coordinated and temporally structured transcriptomic programs that reflect injury-induced remodeling in the dentate gyrus and highlight novel molecular mediators of synaptic reorganization, neuroinflammation, metabolic adaptation, and compensatory plasticity following entorhinal denervation.

## Introduction

Brain injury causes local damage as well as secondary denervation damage in areas connected to the lesion site. Denervated areas exhibit dynamic cellular alterations, including early functional adaptations, glial cell responses to the injury, collateral sprouting of non-lesioned axons, reinnervation of surviving neurons, and dendritic remodeling (Steward, 1989; Steward, 1994; Deller and Frotscher, 1997; Deller et al., 2006; Perederiy and Westbrook, 2013).

A well-established model system for studying lesion-induced neuronal circuit reorganization in the CNS is the denervation of the dentate gyrus following unilateral entorhinal cortex lesion (ECL) (Lynch and Cotman, 1975; Steward, 1994; Deller and Frotscher, 1997; Perederiy and Westbrook, 2013). Using this model, several candidate molecules have been identified that may regulate the reorganization of the dentate gyrus following lesion, including neurotrophic factors, cell adhesion molecules, and extracellular matrix molecules. However, most analyses were limited to a few time points and a single or very few molecules (Poirier et al., 1991; Haas et al., 1997; Deller et al., 2000; Jensen et al., 2000; Poulsen et al., 2000; Thon et al., 2000; Collazos-Castro and Nieto-Sampedro, 2001; Ying et al., 2001; Ying et al., 2002; Burbach et al., 2003; Dong et al., 2004; Ying et al., 2004; Dong et al., 2005; Liu et al., 2005; Wang et al., 2005; Wang and Zhou, 2005; Dehn et al., 2006; Dong et al., 2006; Ginsberg, 2010; Del Turco et al., 2014; Schlaudraff et al., 2022; Del Turco et al., 2023). Thus, a more comprehensive picture of what is happening in denervated brain regions over time is still lacking. This global view is essential, however, to understand the time sequence of the denervation-induced adaptations, their interdependencies, and their overall effects on neuronal function.

To obtain a more complete picture of denervation-induced changes at the transcriptional level, we (i) analyzed changes at critical time points post-lesion, (ii) used laser microdissection to selectively harvest the denervated outer molecular layer (OML) and the granule cell layer (GCL) of the dentate gyrus, and (iii) used microarrays to identify transcriptomic changes. In extension of our previous work, which used subsets of the microarray data (Del Turco et al., 2014; Del Turco et al., 2023), we have now analyzed the transcriptomic landscape. This global approach, which considered layer-specific changes in regulatory molecules over time, revealed several pathways that had not yet been considered relevant in the context of denervation-induced network remodeling. Exploring these pathways further may lead to new targets for drug development.

## Materials and methods

### Entorhinal denervation

As previously published (Del Turco et al., 2014), ~12-week-old male C57BL/6 J mice (Janvier Labs, Le Genest-Saint-Isle, France) were used for unilateral transection of the left perforant path using a wire knife (David Kopf Instruments, Tujunga, CA). The total surgical procedure required approximately 30–45 min per animal. Experimental animals were allowed to survive for 1, 3, 7, 14, or 28 days post-lesion (dpl). Initially, six to seven animals were operated on per time point. Subsequently, three lesioned animals per time point, as well as three non-lesioned (control) animals, were used for microarray analysis.

### Tissue preparation and lesion controls

Tissue preparation and lesion controls were performed as previously described (Del Turco et al., 2014). In brief, mice were euthanized with an overdose of isoflurane, and their brains were removed from the cranium, embedded in tissue freezing medium (Leica Microsystems, Bensheim, Germany), and flash-frozen for 2 min in −70 °C isopentane before being stored at −80 °C until further processing. For the earliest time point (1 dpl), the lesion site could be visualized using toluidine blue staining on horizontal brain sections. For later time points (3–14 dpl), entorhinal denervation was visualized on coronal brain sections using Fluoro-Jade C staining (Histo-Chem, Jefferson, USA). For the latest time point (28 dpl), immunostaining for the astrocytic marker glial fibrillary acidic protein (GFAP) was used to visualize the denervation-induced glial reaction.

### Laser microdissection and RNA isolation

Laser microdissection (LMD) and RNA isolation were performed as previously reported (Del Turco et al., 2014). Briefly, 16-μm-thick coronal brain sections were cut with a cryostat (Leica Microsystems, Nussloch, Germany) and mounted on polyethylene naphthalene (PEN) membrane slides (Leica Microsystems, Wetzlar, Germany). Sections were dried for 2 min at room temperature (RT), fixed in −20 °C cold 75% and 100% ethanol (AppliChem, Darmstadt, Germany) for 2 min each, and stored at −80 °C until further processing. For LMD, sections were thawed, stained with a 1% cresyl violet solution (Sigma-Aldrich, St. Louis, USA) at RT for 15–30 s, and briefly dehydrated in 75% and 100% ethanol. A Leica 6500 LMD system (Leica Microsystems) was used to cut defined tissue portions of the granule cell layer and the outer molecular layer using a 20x objective. Tissues were collected in separate tubes containing 50 μL of lysis buffer (RNeasy Plus Micro Kit; Qiagen, Hilden, Germany) supplemented with ß-mercaptoethanol (Sigma-Aldrich). Tubes were refilled to 350 μL with RLT lysis buffer (RNeasy Plus Micro Kit), vortexed for 30 s, and transferred immediately to −80 °C until further processing. Total RNA was isolated using the RNeasy Plus Micro Kit (Qiagen) according to the manufacturer’s recommendations. Prior to microarray processing, RNA integrity (RIN > 8) was assessed using the Agilent Bioanalyzer system and RNA 6000 Pico Kit (Agilent Technologies, Waldbronn, Germany).

### Microarray analysis

For microarray analysis (see Del Turco et al., 2014), total RNA was transcribed into cDNA and amplified using single primer isothermal amplification (SPIA) technology with the Ovation® Pico WTA System (NuGEN, AC Bemmel, Netherlands). Next, the Encore Biotin Module Kit (NuGEN) was used for fragmentation and labeling of cDNA for analysis on Affymetrix GeneChips®. Samples were hybridized on a Mouse Gene 1.0 ST array (Affymetrix, High Wycombe, United Kingdom). All steps were performed following the manufacturer’s recommendations via MFT Service (Tübingen, Germany). Raw data were checked for technical quality using Expression Console software (Affymetrix). For downstream analysis, CEL files were normalized using the Robust Multi-Array Average (RMA) algorithm in Partek Genomic Suite 6.5 (PGS, Partek, Chesterfield, USA) with default settings. Possible batch effects, for example due to arrays being scanned on different dates, were controlled using the batch removal tool in PGS. Despite this correction, one animal had to be excluded from further analyses due to technical problems during hybridization. The selection cutoff for differentially expressed genes was set to a *p*-value of ≤ 0.05 based on t-statistic values. Previous studies reported only small subsets of the microarray data (Del Turco et al., 2014; Del Turco et al., 2023). To enable future studies on other topics, all original microarray data were deposited publicly at the Gene Expression Omnibus (GEO) repository, with the accession numbers GSE284333 (GCL) and GSE284334 (OML). The original computations documented raw fold changes, and Tables 1–4 and supplements maintain raw FC because neuronal transcripts show much milder dysregulations than glial transcripts, making it easier to distinguish mild reductions due to tissue loss from credible downregulations via color grading in heatmaps in non-logarithmic scales. In contrast, the overview of particularly interesting factors in the final summary figure uses log_2_ fold changes, where the much stronger dysregulations of glial factors become conspicuous.

To compare gene expression changes across different time points after the lesion, UpSet plots were used for visualization, separately for OML and GCL. UpSet plots were generated using the R package “UpSetR”. Complete gene expression results exported from Partek Genomics Suite are provided as Supplementary Table S1.

### Gene Set Enrichment Analysis (GSEA)

The Gene Set Enrichment Analysis (GSEA; Broad Institute; Cambridge, MA) approach was used to identify defined networks of gene interactions using curated gene sets from the Molecular Signature Database (MSigDB 2023.2.Mm). All whole-transcript array data from the OML and the GCL for each time point were pre-ranked by fold change (without any filtering by significance) and then subjected to analyses with default settings. A normalized enrichment score was calculated for each gene set to represent the degree to which it was enriched in the tested phenotype, and results were filtered with a family-wise error rate (FWER) *p*-value < 0.05. GSEA in the GCL detected over-representation for a myriad of significant pathways, particularly for upregulated biological processes, so intrinsic validation was imposed, listing only effects in GSEA-illustrating figures that were reproduced at another time point. This detailed output offers the opportunity to relate the overall pathway changes to a few large and many small expression changes of specific transcripts. To visualize gene set enrichment results across different time points after lesion, GSEA results were summarized using heatmaps based on enrichment scores (ES). Heatmaps were generated in R using the “pheatmap” package. The comprehensive GSEA approach for Gene Ontology (GO) gene sets for the individual time points after denervation is provided in detail in Supplementary Tables S2–S7.

### Search tool for retrieval of interacting genes/proteins (STRING)

The STRING (Search Tool for the Retrieval of Interacting Genes/Proteins; at the European Molecular Biology Lab, Heidelberg, Germany) approach was used (https://string-db.org/, last accessed on August 2025) to identify defined networks of gene interactions using curated gene sets from the KEGG (Kyoto Encyclopedia of Genes and Genomes at the Institute for Chemical Research, Kyoto University, Uji, Kyoto, Japan) and from the Reactome (Ontario Institute for Cancer Research, Toronto, Canada) databases. Factors with nominally significant down- versus upregulations from the OML and the GCL for each time point were used as input (without any filtering by fold changes) and then subjected to analyses with default settings. A normalized enrichment score was calculated for each gene set to represent the degree to which it was enriched in the tested phenotype, and the ranking occurred by false discovery rates (FDR). Corresponding KEGG and Reactome pathway enrichment results for OML and GCL at individual time points after denervation are provided in Supplementary Files S1, S2.

### Curated analyses of heatmap tables on the basis of public databases

Nominal significances served as the basis for further pathway and interaction enrichment analysis on the STRING website, where multiple testing artifacts are controlled by false discovery rate (FDR) analysis. To understand the features and roles of individual factors, the GeneCards, UniProt, PubMed databases, and the Allen mouse *in situ* hybridization (ISH) atlas were interrogated. The neural expression of each listed factor was assessed in the literature or in a cell-type selective ribonucleic acid sequencing (RNAseq) (high-throughput, next-generation) database (www.braincellatlas.org/, last accessed on March 2025). Most factors are expressed in all dentate neurons or preferentially in granule cells, according to the Allen mouse brain atlas ISH pattern. Where this is not the case, the expression of the factors in interneurons or in specific glia or immune cell types was noted.

The RNAseq database https://public.brain.mpg.de/dashapps/localseq/explore (last accessed on in October 2025), where rat hippocampal CA1 area transcriptomes of dissected pyramidal somata/axons versus dissected molecular layer neuropil (with dendrites, postsynapses, and glia) were deposited, was further used to understand in an unbiased manner whether an individual mRNA is preferentially transported to axons, dendrites, or all types of neurites.

Due to the elimination of one animal where technical problems arose during the microarray hybridization, the statistical power at 7 dpl was particularly low, explaining the lack of significant dysregulation at this time point for transcripts with otherwise consistently perfect chronic expression changes, such as the down regulations of *Mtap2*, *Gabra1*, and the upregulations of *Smn1* and *Dlg4* in the GCL. Data processing and statistical analyses were conducted in R (version 2026.01.0 Build 392; R Core Team). To assess consistent significance across multiple time points, *p*-values were aggregated using the harmonic mean p-value (HMP) approach (Wilson, 2019), which is robust to heterogeneity among tests. HMP values were computed across five time points per gene. The heatmap was generated with GraphPad Prism software, version 10 (GraphPad Software, Boston, MA, USA).

## Results

**Figure 1:**
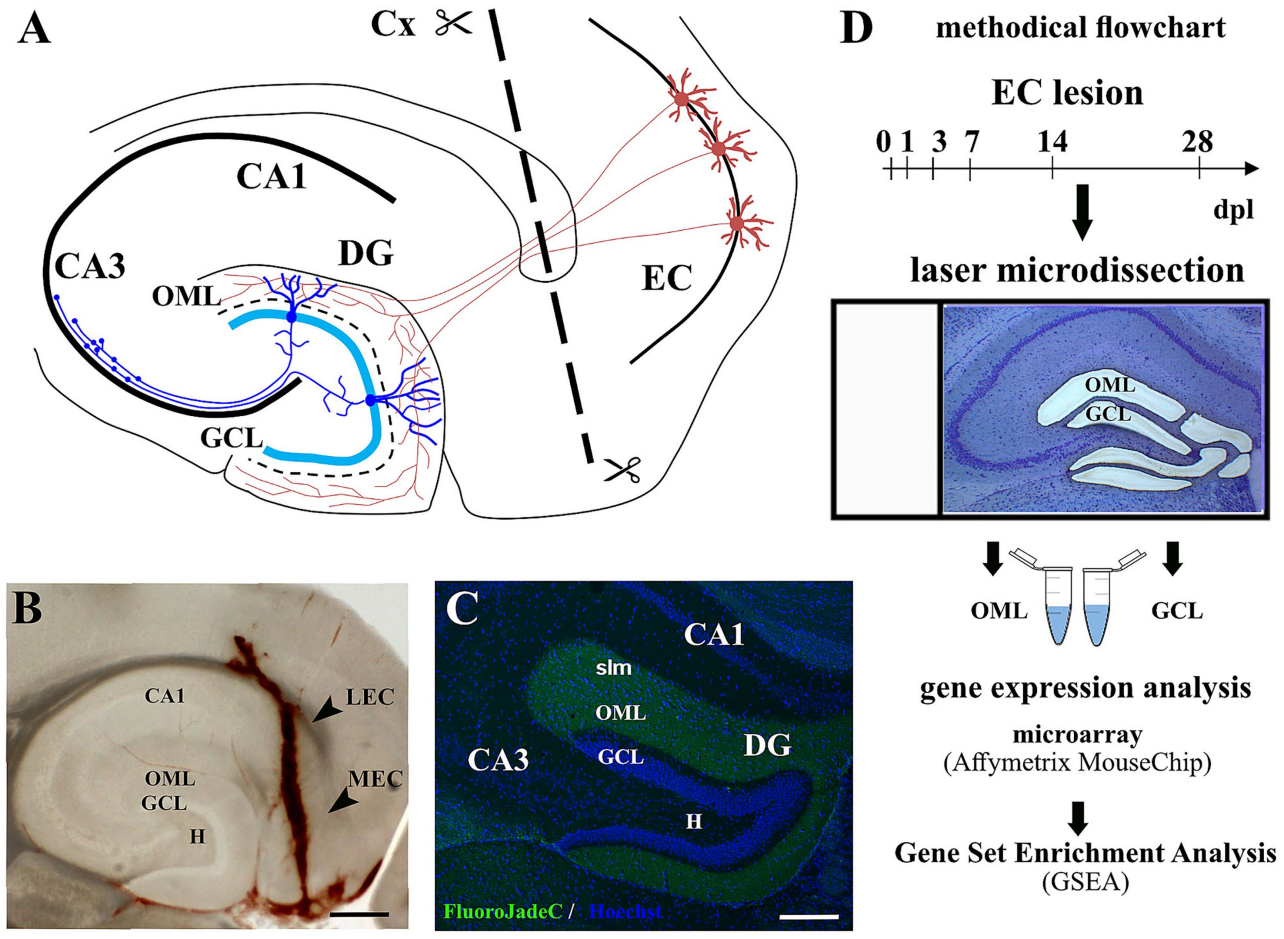
Entorhinal denervation model used for gene expression study of two dentate subregions after denervation. **(A)** Schematic hippocampus illustrates the model of entorhinal denervation. The perforant path (red lines) originates from stellate neurons in the lateral and medial entorhinal cortex (LEC + MEC = EC) and terminates on distal parts of the granule cell dendrites in the outer molecular layer (OML) of the dentate gyrus (DG). The transection of the perforant path is indicated by the line (Del Turco et al., 2014). **(B)** A representative brain section (horizontal plane) one day after the lesion verified the correct site of lesioning (arrowheads). **(C)** Layer-specific denervation of the OML could be visualized via Fluoro-Jade C staining in green. **(D)** The methodical workflow summarizes the main steps. Animals were analyzed at different time points after entorhinal denervation (days post lesion, dpl). After Nissl staining with Cresyl violet, laser microdissection was used to isolate tissue from the granule cell layer (GCL) and the OML separately. Whole-genome expression analysis was performed with Affymetrix Gene Chip (Mouse Gene 1.0 ST array). Gene Set Enrichment Analysis (GSEA) and STRING pathway enrichment studies compared expression changes with predefined gene sets. CA1/CA3: Cornu Ammonis subregions; Cx: cortex; H: hilus; slm: stratum lacunosum moleculare. Scale bars: 250 μm.

In view of the large number of neuron-expressed dysregulated factors in this project, and the need to consider the biochemical features, subcellular distribution, and functional roles of each to understand their interactions in co-regulated pathways, a comprehensive compilation of data regarding each transcript change is provided in Supplementary Table S1. Key findings offering meaningful insights into the temporal and spatial neuronal efforts to compensate for the injury are provided in the main Results section below, from the experimental design in Figure 1 to the Graphical Abstract of findings.

**Figure 2:**
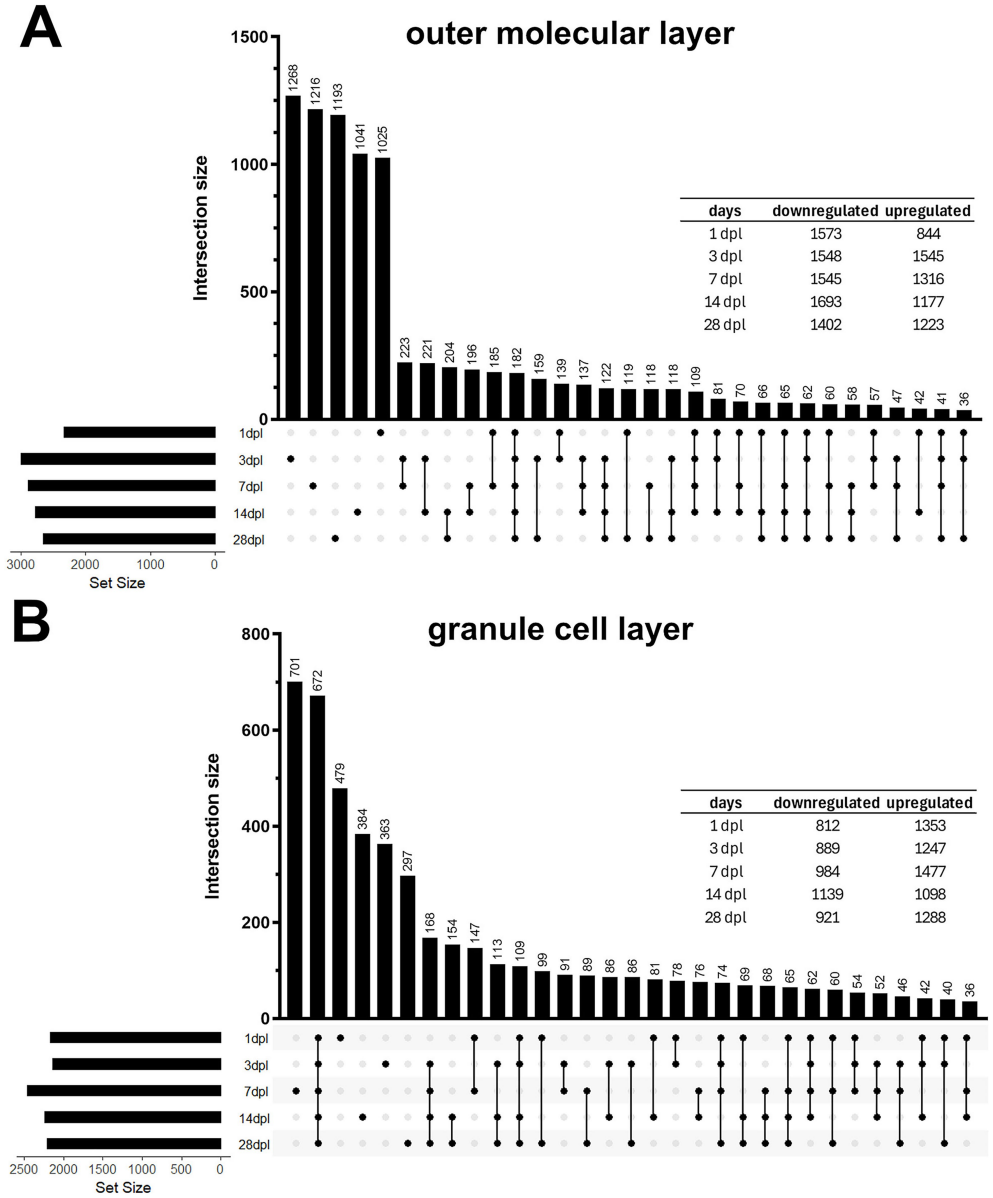
Number of down- and upregulations, with UpSet plot of expression changes in the dentate OML and GCL. A high number of transcripts were dysregulated over five time points after entorhinal lesion in OML **(A)** as well as in the GCL **(B)**. UpSet plots showed that most transcripts changed at only one time point, and that exclusively in the GCL a high number of transcripts exhibited dysregulation at all time points. Exact numbers of down- and upregulated genes are shown in tabular form in the right part of the figure panels for each time point.

The analysis of expression changes across individual time points showed rapid responses already at 1 dpl, with a peak in the number of dysregulations at 3 dpl in the OML and at 7 dpl in the GCL (Figure 2). The number of transcript adjustments was reduced to half at 28 dpl in the OML but was still ongoing in the GCL. Many upregulations in the OML reflected gliosis. The fold change (FC) of expression regulations was smaller at 1 dpl in both regions, peaked for upregulations at 3 dpl in the OML (mainly macrophage and microglia factors), but remained quite constant afterwards in both the OML and GCL. UpSet plots for the OML showed significantly fewer dysregulated transcripts with consistency for each time point than for the GCL, suggesting more dynamic transcription programs in the OML over time (likely reflecting repair), while GCL stress responses exhibited a more uniform pattern (as permanent adaptations).

### I. Transcriptional adjustments occurring in the OML

**Figure 3:**
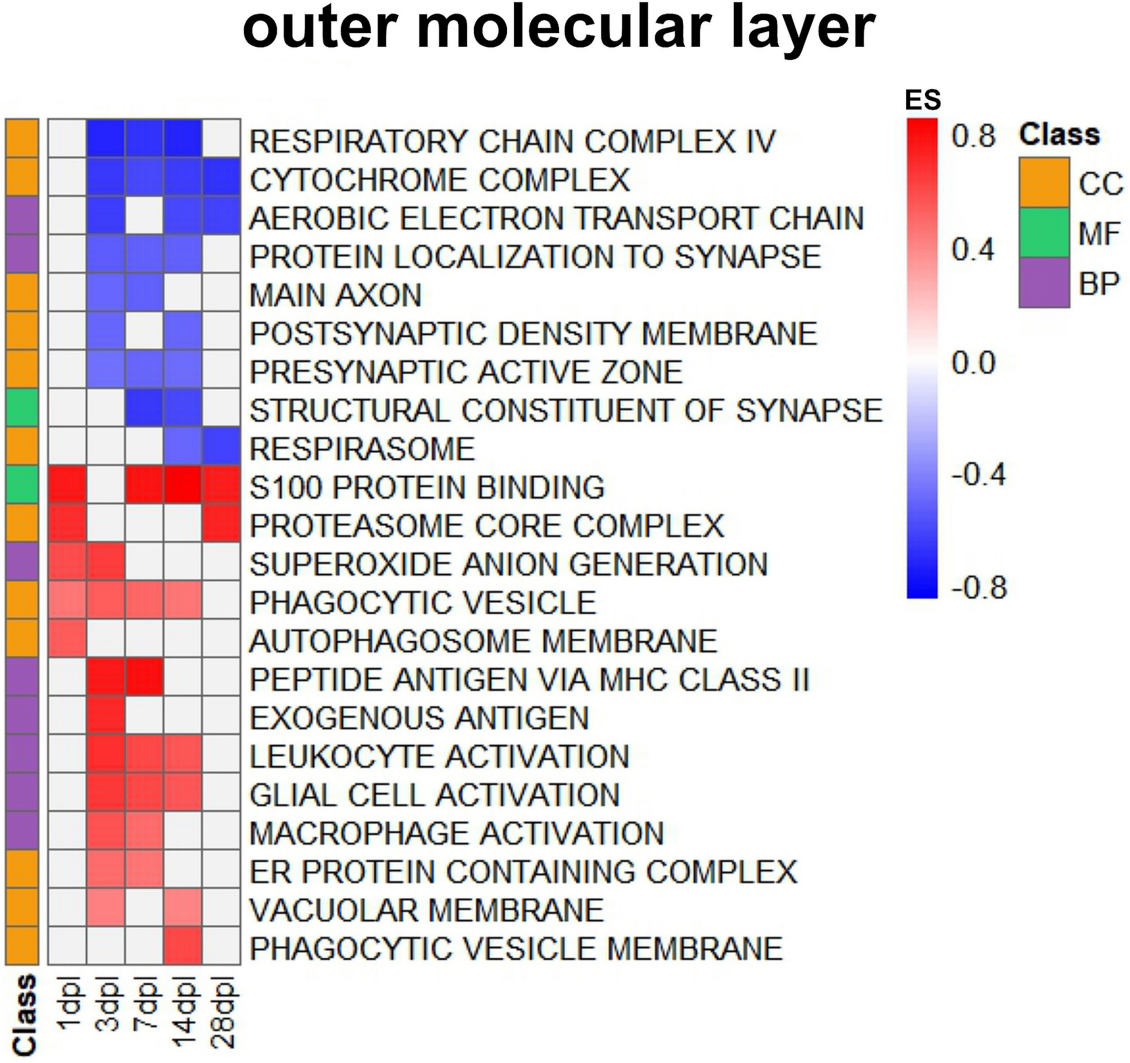
Pathway enrichment analysis via GSEA, showing GO terms in the dentate OML after entorhinal lesion. Transcripts ranked by fold change were analyzed using Gene Set Enrichment Analysis (GSEA) software. The heatmap displays significantly enriched Gene Ontology (GO) terms in the outer molecular layer at different time points after the lesion (dpl). Rows correspond to GO terms from the categories Cellular Component (CC, orange), Molecular Function (MF, green), and Biological Process (BP, violet), as indicated by the color-coded class bar on the left. The color intensity of the heatmap reflects the enrichment score (ES), with red indicating positive and blue indicating negative enrichment.

GSEA filtering of OML data (Supplementary Tables S2–S4) emphasized that entorhinal denervation first triggered downregulations (Figure 3) from 3 dpl onwards for the neurotransmission machinery (GOCC terms for postsynaptic density, main axon, presynaptic active zone) and neuronal respiration (GOCC terms for cytochrome complex, respiratory chain complex IV, respirasome, exemplified in Table 1 by *Cox6a1* and *Cox1* decreases), in an acute and sustained manner. Structural constituents of synapses (GOMF term) were in diminished demand during the 7–14 dpl peak. Protein localization to the synapse and aerobic electron transport chain (GOBP terms) appeared impaired since 3 dpl.

The entorhinal denervation secondly triggered upregulations (Figure 3) of protein/RNA/lipid degradation efforts (GOCC terms of proteasome core complex, phagocytic cycle, phagocytic vesicle membrane, autophagosome membrane, endoplasmic reticulum protein-containing complex, vacuolar membrane), in an acute and sustained manner mostly until 14 dpl. These efforts are performed primarily by microglia, with the assistance of additional invading macrophages. They occurred in parallel to the GOMF term for S100 protein binding, which represents well-known glial markers of damage in the nervous system (Bottiger et al., 2001). GOBP terms illustrated that the activation of glia and leukocytes was accompanied by the generation of superoxides (presumably during oxidative bursts of macrophages and microglia while they engulf toxic debris), elevated antigen presentation (presumably to assess the presence of foreign bodies or microorganisms among the breakdown products), as well as leukocyte/macrophage/glia cell activations at 3–14 dpl. These findings, which indicate reactive changes of various cell types (in particular glia), the activation of degradation and repair programs, altered neuronal signaling, and overall energy consumption, are expected and align with current concepts of brain repair.

Complementary enrichment studies across the transcriptomes via the STRING Heidelberg tool, regarding the pathways compiled in the KEGG and Reactome databases, confirmed these effects (Supplementary File S2). In addition, an impact on apoptosis and hemostasis was apparent from 1 to 14 dpl.

In the following paragraphs with subtitles, a stratification within this “big data” landscape was attempted, subgrouping the transcript changes according to neuronal compartments, temporal co-regulations, and functional pathways to maximize the recognition of co-regulated patterns.

Manual selection of significantly changed mRNAs was conducted, adhering to the following criteria: (i) intrinsic validation upon significant effect reproduction at an adjacent time point; (ii) FC above 1.15 or below 0.87 for enhanced pathway recognition; (iii) published roles for shaping neuronal subcompartments, if possible, in hippocampal studies. This focus on changes in neuronal morphology, relating them to underlying waves of transcription programs, cannot be exhaustive within one manuscript. It will overlook short transient transcription patterns and anonymous mRNAs, as well as important aspects of glial contribution, excitability, and metabolism, particularly by neglecting ion and solute trafficking factors. The compilation of selected transcripts in the main Tables 1–4 used different color hues (red for upregulations, blue for downregulations) within a heatmap representation of FC and nominal significances with *p*-values (in orange hues) for each factor and time point. This illustration allows readers to quickly survey which differential regulations are prominent against a backdrop of reductions where neuronal tissue loss and dedifferentiation act as confounders, and an induction backdrop where microgliosis and astrogliosis may contribute. To enable relative comparisons to neuronal tissue loss and gliosis gain, compartmental markers were included, first for axon markers such as *Sema3e/Nefl/Nefm/Nefh* (Table 1), second for astrocytes such as *Gfap/Vim*, and third for microglia such as *Aif1/Tyrobp* (Table 2). To visualize temporal co-regulations, factors are grouped first by acute transient dysregulations, then chronic changes, and finally delayed and terminal responses. Within such clusters, factors were ordered by their relevance for the overall reprogramming of postsynapse – dendrite – soma – axon – presynapse. Columns from left to right indicate the gene symbol and transcript identity number within the microarray, for each time point, the p-value, and the FC of expression adjustment. Factors with prominent and consistent effect sizes in FC were highlighted in bold.

**Table 1:** Downregulated transcripts in dentate OML after entorhinal lesion.

Gene symbol	Transcript ID	1 dpl *p*-value	1 dpl FC	3 dpl *p*-value	3 dpl FC	7 dpl *p-*value	7 dpl FC	14 dpl *p*-value	14 dpl FC	28 dpl *p*-value	28 dpl FC	HMP
Sema3e	10519747	0.05	0.73	0.05	0.73	0.01	0.60	0.05	0.73	0.20	0.80	0.03
Ppfia2	10366196	7.48E-04	0.48	2.30E-03	0.54	9.35E-03	0.58	0.04	0.69	0.13	0.75	0.00
Calb1	10503416	4.73E-03	0.40	0.01	0.47	0.05	0.54	0.19	0.70	0.81	0.93	0.02
Sphkap	10356154	1.43E-03	0.53	1.86E-03	0.54	0.01	0.60	0.10	0.76	0.11	0.75	3.73E-03
Irx4	10406017	7.60E-05	0.80	0.03	0.92	0.04	0.91	4.62E-04	0.84	0.34	0.96	3.25E-04
Bhlhe22	10490950	2.19E-04	0.58	8.44E-03	0.73	0.06	0.79	0.04	0.79	0.18	0.85	1.06E-03
Gabra1	10385297	0.02	0.65	9.94E-03	0.61	0.12	0.74	1.14E-03	0.49	0.16	0.76	4.79E-03
Sema5a	10423520	0.01	0.79	0.11	0.88	0.02	0.80	0.33	0.93	0.49	0.94	0.03
Mageb4	10605598	5.75E-04	0.84	0.06	0.93	0.15	0.94	0.51	0.98	0.80	1.01	2.83E-03
Unc13a	10579554	5.43E-03	0.78	1.75E-05	0.58	1.04E-03	0.70	8.29E-03	0.79	9.80E-03	0.78	8.55E-05
P2rx2	10532346	6.65E-04	0.86	8.48E-03	0.90	0.01	0.90	5.00E-03	0.89	0.02	0.91	2.57E-03
Cntn4	10540359	0.02	0.69	6.75E-03	0.65	0.03	0.70	8.10E-03	0.65	9.84E-03	0.63	0.01
Gad1	10472707	3.92E-03	0.70	1.16E-04	0.56	1.64E-04	0.54	1.52E-03	0.66	0.01	0.73	3.18E-04
Nefl	10416175	1.21E-03	0.81	1.05E-04	0.74	1.44E-03	0.79	1.52E-05	0.69	1.58E-05	0.66	3.57E-05
**Adamts3**	10531195	1.18E-05	0.14	1.14E-05	0.14	3.47E-05	0.15	5.27E-05	0.19	3.29E-04	0.23	2.24E-05
Btbd3	10476538	2.61E-03	0.71	3.10E-03	0.71	4.61E-03	0.70	9.91E-03	0.76	0.04	0.80	4.77E-03
Agtpbp1	10409737	0.01	0.80	1.59E-03	0.73	4.11E-03	0.74	3.89E-04	0.68	0.02	0.79	1.39E-03
Cnih2	10476538	0.01	0.70	4.04E-03	0.64	6.48E-04	0.52	8.89E-05	0.47	1.44E-03	0.55	3.62E-04
Neto1	10457091	0.03	0.61	8.78E-04	0.39	0.03	0.55	0.02	0.56	0.04	0.59	3.89E-03
Rxfp1	10498852	3.45E-03	0.86	0.01	0.88	7.38E-04	0.81	0.01	0.89	4.51E-03	0.85	2.47E-03
Vamp1	10541877	0.02	0.58	4.21E-03	0.49	2.54E-04	0.31	0.03	0.62	5.71E-03	0.47	1.13E-03
Dlg2	10554900	0.01	0.82	7.78E-03	0.80	5.94E-03	0.77	2.63E-03	0.77	0.03	0.83	0.01
Snap91	10595496	3.17E-03	0.79	2.10E-04	0.70	0.02	0.82	0.05	0.87	0.04	0.85	9.67E-04
Lypd6	10472034	6.65E-04	0.53	0.05	0.74	1.03E-03	0.51	6.07E-03	0.64	8.97E-04	0.51	1.32E-03
Grm8	10543494	5.44E-03	0.69	1.25E-03	0.63	2.39E-03	0.62	1.74E-03	0.64	7.40E-03	0.68	2.37E-03
Grm7	10540509	8.57E-03	0.70	0.17	0.85	0.01	0.69	0.03	0.76	6.42E-03	0.65	0.01
Nps	10558400	6.24E-04	0.75	0.86	0.99	0.02	0.82	0.01	0.83	0.03	0.84	2.79E-03
Lrrc7	10503054	4.96E-03	0.82	0.19	0.92	0.00	0.79	0.03	0.86	0.28	0.93	0.01
Ptpn3	10513160	0.04	0.36	0.09	0.45	0.03	0.30	0.03	0.34	0.07	0.39	0.04
Kalrn	10439138	2.00E-03	0.75	2.05E-03	0.75	2.14E-03	0.73	0.02	0.82	0.09	0.87	3.27E-03
Kif5a	10373113	0.04	1.23	9.73E-05	0.58	9.61E-05	0.55	3.42E-03	0.72	0.02	0.76	2.38E-04
Kif5c	10471994	0.92	0.99	2.21E-03	0.68	9.13E-04	0.61	3.93E-03	0.70	0.19	0.86	2.76E-03
Vcan	10410931	0.22	0.81	0.02	0.63	0.04	0.66	0.01	0.59	0.59	0.91	0.02
Nefm	10421100	0.07	0.68	7.66E-04	0.40	1.57E-03	0.40	4.80E-03	0.50	0.08	0.65	2.29E-03
Dock3	10596583	0.89	0.98	0.03	0.65	0.02	0.58	0.05	0.68	0.12	0.73	0.04
Stxbp1	10481711	0.06	0.72	0.09	0.75	7.97E-03	0.56	0.02	0.65	0.08	0.71	0.02
Nrsn1	10408359	0.19	0.91	8.19E-06	0.58	1.01E-03	0.72	3.37E-06	0.55	3.88E-06	0.52	7.38E-06
Pvalb	10430297	0.06	0.53	0.02	0.46	0.01	0.36	0.03	0.47	0.02	0.39	0.02
Cox6a1	10532993	0.95	1.00	2.31E-06	0.78	1.21E-06	0.74	1.68E-06	0.77	6.22E-07	0.72	1.44E-06
Ddn	10432278	0.05	0.90	2.28E-06	0.62	2.08E-07	0.50	3.65E-07	0.56	1.93E-05	0.66	6.22E-07
Trim32	10505512	0.29	0.95	4.87E-06	0.66	0.19	1.08	6.85E-03	0.85	0.49	0.96	2.44E-05
Homer2	10565152	0.30	0.92	4.56E-05	0.59	0.75	0.97	2.75E-04	0.65	5.43E-05	0.56	1.14E-04
Hapln4	10572282	0.20	0.88	0.03	0.79	6.45E-03	0.70	0.09	0.84	0.06	0.80	0.02
Grm3	10528145	0.13	0.77	1.68E-03	0.52	0.06	0.69	0.33	0.85	0.01	0.57	0.01
Gria1	10376245	0.09	0.85	0.15	0.87	0.04	0.80	0.01	0.77	0.78	1.03	0.04
Nrn1	10408689	0.08	0.67	0.24	0.77	0.02	0.53	0.01	0.48	0.36	0.80	0.02
Eef1a2	10490602	0.43	0.93	0.09	0.85	0.00	0.70	7.02E-03	0.75	0.04	0.80	0.01
Dnalc1	10397179	0.20	0.69	0.06	0.57	0.02	0.44	0.02	0.47	0.04	0.49	0.03
Nefh	10383920	0.11	0.86	0.41	0.93	2.02E-03	0.67	1.36E-03	0.69	9.10E-03	0.73	3.70E-03
Prkcz	10519117	0.06	0.80	0.08	0.82	0.01	0.71	0.13	0.84	0.04	0.77	0.04
Cox1	10598036	0.95	1.00	0.06	0.94	0.68	0.99	1.27E-03	0.88	7.40E-05	0.81	3.49E-04
Dgki	10543967	0.15	0.79	0.17	0.80	0.29	0.83	2.45E-03	0.55	0.02	0.63	0.01
Fat3	10591135	0.62	0.92	0.27	0.82	0.27	0.80	1.09E-03	0.47	0.04	0.65	0.01

**Table 2:** Upregulated transcripts in hippocampal OML after entorhinal lesion.

Gene symbol	Transcript ID	1 dpl *p*-value	1 dpl FC	3 dpl *p*-value	3 dpl FC	7 dpl *p*-value	7 dpl FC	14 dpl *p*-value	14 dpl FC	28 dpl *p*-value	28 dpl FC	HMP
Atox1	10386070	0.08	1.25	1.05E-04	1.99	0.25	1.16	0.02	1.38	0.12	1.24	5.21E-04
Ftl1	10447591	0.02	1.33	5.46E-05	1.91	7.10E-03	1.44	4.35E-03	1.43	0.01	1.38	2.66E-04
Jun	10514466	8.72E-03	1.28	8.65E-03	1.28	2.45E-04	1.59	1.79E-04	1.54	8.36E-05	1.70	2.29E-04
S100a6	10493820	3.17E-03	1.50	4.47E-05	2.06	3.90E-05	2.27	9.16E-06	2.38	7.50E-04	1.76	3.15E-05
Arl4c	10356475	4.96E-05	1.51	5.89E-06	1.70	1.38E-04	1.51	1.08E-04	1.46	1.28E-03	1.36	2.41E-05
Gabarap	10377689	6.17E-03	1.27	8.25E-05	1.56	6.46E-04	1.46	1.24E-03	1.36	1.02E-03	1.43	3.20E-04
Tesc	10524955	1.69E-08	2.16	1.13E-06	1.64	6.17E-05	1.42	2.48E-04	1.30	3.33E-06	1.64	8.27E-08
Serpina3n	10398075	1.92E-04	3.26	6.41E-05	3.88	2.06E-06	9.46	2.94E-07	11.94	9.41E-05	4.25	1.28E-06
Sparc	10386058	2.59E-03	1.32	5.03E-06	1.84	2.81E-05	1.75	1.49E-05	1.72	1.76E-03	1.39	1.65E-05
Gfap	10391798	3.73E-04	1.77	1.42E-06	2.99	1.18E-06	3.49	6.21E-07	3.32	5.05E-06	2.90	1.49E-06
Vim	10469322	2.42E-03	1.43	2.08E-04	1.65	1.17E-05	2.21	4.31E-06	2.21	6.51E-05	1.91	1.48E-05
Aif1	10450484	0.85	0.97	4.94E-04	2.55	0.01	1.88	0.03	1.61	0.23	1.30	2.33E-03
Tyrobp	10551883	5.01E-03	1.30	8.54E-07	2.17	5.75E-06	2.02	1.23E-05	1.78	3.88E-04	1.53	3.50E-06
Glycam1	10433172	0.02	2.46	2.36E-05	11.86	3.70E-04	7.16	1.76E-04	6.95	3.60E-03	4.12	9.78E-05
Gsn	10471655	0.21	1.18	0.03	1.37	3.75E-03	1.66	0.01	1.44	0.04	1.38	0.01
Mafb	10489246	0.10	1.28	1.14E-05	2.95	5.23E-03	1.71	4.50E-03	1.63	8.60E-03	1.63	5.69E-05
Rab8b	10594645	0.10	1.42	0.01	1.83	0.04	1.67	0.07	1.47	0.05	1.61	0.03
Cxcl5	10523120	0.34	1.19	1.45E-05	3.74	2.55E-05	3.98	5.92E-05	3.06	4.06E-04	2.66	3.93E-05
Cdk5rap2	10513824	0.24	1.15	0.07	1.27	3.65E-02	1.36	1.14E-03	1.68	2.30E-03	1.69	3.68E-03
Cst3	10488415	0.07	1.05	0.70	0.99	1.23E-03	1.13	1.06E-04	1.17	8.07E-03	1.10	4.79E-04
B2m	10475414	0.08	1.18	3.39E-04	1.58	3.85E-04	1.65	5.78E-04	1.53	0.29	1.11	6.86E-04
Ccr5	10590635	0.33	1.31	0.03	1.93	2.29E-03	3.28	0.02	2.02	0.09	1.74	0.01
Cyfip1	10553598	0.89	0.99	4.86E-03	1.41	2.23E-03	1.55	0.07	1.22	0.85	1.02	0.01
Nrp1	10576639	0.54	1.12	0.02	1.60	0.04	1.57	0.03	1.53	0.23	1.29	0.05
Tmsb4x	10607865	0.58	1.03	1.72E-04	1.39	0.03	1.18	0.15	1.09	0.60	0.97	8.53E-04
Arf4	10413304	0.68	1.07	0.08	1.40	0.03	1.64	0.03	1.55	0.06	1.49	0.05
Npas3	10395733	0.21	1.28	0.06	1.47	0.04	1.61	0.02	1.67	0.54	1.14	0.05
Lyn	10503098	0.06	1.45	1.46E-03	2.12	0.05	1.55	1.73E-03	2.08	0.20	1.30	3.83E-03
Apoe	10560624	0.13	1.11	0.53	0.96	0.72	1.03	6.31E-03	1.23	6.31E-03	1.27	0.02

Immediate effects of entorhinal afferent loss in the shrinking OML tissue (Table 1) were exemplified by significant reductions at 1–14 dpl, with a minimum around 60% at 7 dpl, and normalization by 28 dpl, for *Sema3e* mRNA (encoding the secreted ligand semaphorin 3E) as their axon repellent (Pozas et al., 2001). Similarly, a reduction of about 80% at 1–7 dpl for *Sema5a* (encoding the transmembrane ligand semaphorin 5A) as a granule cell dendritic spine biogenesis factor was documented (Duan et al., 2014). Thus, an immediate marked impact on axon guidance cues was prominent.

The proportional gain of glia in OML tissue (Table 2) was reflected, e.g., by significant chronic inductions of *Tyrobp* (1.3–2.2-fold) and *Aif1* (1.6–2.6-fold) as microglia markers with a maximum at 3 dpl, as well as *Gfap* (1.8–3.5-fold) and *Vim* (1.4–2.2-fold) as astrocyte markers with a maximum at 7 dpl. Tables 2, 4 were selected to include meaningful neuronal upregulations rather than gliosis factors.

An interesting pattern involved the GCL induction of *Tcfeb* mRNA (encoding transcription factor TFEB), which controls the expression of lysosomal biogenesis and autophagy factors (Settembre et al., 2011), during the initial period of 1–7 dpl when debris must be removed. Subsequently, in the late period of 7–28 dpl, the OML upregulation of *Cst3* (encoding a cysteine protease inhibitor) suggests that debris breakdown has ceased, while simultaneous decreases in the neuronal, stimulus-dependent translation elongation factor *Eef1a2* indicate that protein biosynthesis remains limited (McLachlan et al., 2019).

Interestingly, the acute transient OML downregulation of *Mageb4* at 1–3 dpl, a factor with exclusive expression in non-proliferative stem cells (Osterlund et al., 2000), may suggest a neurogenesis response already at this early stage. Later, at 7–28 dpl, the induction of *Notch2* as a neurogenesis repressor (Engler et al., 2018) implies that proliferation has concluded and differentiation has commenced.

#### Remodeling response of OML glia activators and repressors

Specific glial factors were crucial for the reabsorption of damaged tissue. During peak repair at 3–14 dpl, macrophage/glia-expressed *Atox1* was elevated, reaching a maximum at 3 dpl, as a factor responsible for copper removal from debris (Zhao et al., 2024). In contrast, a chronic increase peaking at 3 dpl was noted for *Ftl1*, a microglial factor essential for iron homeostasis (Kumar et al., 2016). The chronic upregulation of *Sparc*, known as an entorhinal denervation-induced factor that modulates glutamate homeostasis (Liu et al., 2005), also appeared to contribute to macrophage invasion. An induction of mainly microglia-expressed *B2m*, with neuroinflammatory roles, was documented at 3 dpl and sustained until 14 dpl. This microglial activation was possibly governed by *Mafb*, a factor responsible for macrophage infiltration and adult microglia colonization after brain damage (Zhang et al., 2025), which exhibited delayed induction with a maximum at 3 dpl and sustained upregulation until 28 dpl.

After tissue breakdown, a prominent novel finding was the strong and chronic upregulation of *Serpina3n* mRNA, a serine peptidase inhibitor produced by neurons and glial cells to control gliosis via the NFkB signaling pathway (Zamanian et al., 2012; Liu et al., 2023), with maximal levels at 14 dpl. As a late induction from 14 dpl, reaching maximal levels at 28 dpl, *Apoe* (encoding Apolipoprotein E) appeared to reflect the final resumption of OML activity.

#### Remodeling response of the OML extracellular matrix

The ECM was rapidly modified in the OML after the lesion, in line with published data (Deller et al., 2000). Entorhinal denervation was previously shown to trigger profound expression changes of ECM factors such as tenascin-C (Deller et al., 2000), which exhibited chronic upregulation here, with levels peaking already at 1 dpl. A massive chronic upregulation of *Glycam1*, peaking at 3 dpl, would modulate the initial adhesion of leukocytes to the regional endothelium, followed by tissue infiltration (Imai et al., 1993).

A strong response to the loss of reelin secretion from entorhinal afferents was evident. Chronic massive downregulation with minimal levels at 3 dpl (to 14%) was documented for *Adamts3* (encoding a secreted metalloproteinase), which is responsible for reelin degradation and therefore would promote sustained reelin signaling, increase neurogenesis, and activate biogenesis and maturation of dendritic spines in newborn granule cells (Bosch et al., 2016; Ogino et al., 2017).

#### Remodeling response of the OML axons and presynapses

The neuronal tissue loss in the denervated OML decreases its original size by 30%–50% (Phinney et al., 2004; Vuksic et al., 2011), and most neuronal transcripts in the OML showed similar decreases. It is noteworthy to mention the slightly progressive reductions of unspecific axon markers such as *Nefl* and the primarily interneuron-expressed *Nefh* (encoding the Neurofilament light and heavy chains), decreasing to 66%–81% at 1–28 dpl and 67%–74% at 7–28 dpl, respectively, as correlates of axonal length. Thus, minor chronic transcript reductions in the OML may not represent selective transcriptional downregulations but rather tissue loss.

The period of lowest synaptic signaling appeared to cover 7–14 dpl, given the minimal reductions of the immediate-early transcript *Nrn1* to ~50% (encoding Neuritin-1 as an activity- and neurotrophin-dependent promoter of axon branching) (Naeve et al., 1997; Shimada et al., 2016). In temporal overlap, reductions to ~30% for *Ptpn3* (crucial for intracellular signal transduction from axon pathfinding receptor tyrosine kinases) (Stoker and Dutta, 1998) mirror the peak neurotrophic depletion in the OML, 1–2 weeks after injury. Somewhat later, at 14–28 dpl, the expression reduction of *Fat3* to 47%–65%, a repressor of neuron migration, axon fasciculation, and dendrite development (Mitsui et al., 2002; Seeler et al., 2023), presumably reflects the need to minimize neurite retraction and unipolarity at this stage.

Downregulations occurred for several modulators of vesicle priming that control neurotransmitter release in the active zone. This included a reduction to 48% for *Ppfia2* (encoding liprin-alpha-2, which ensures presynaptic efficacy in the active zone) (Liang et al., 2021), with downregulation persisting until 14 dpl. Furthermore, this included a functional interactome, with reduced *Stxbp1* to ~60% (encoding Munc18-1, primarily expressed in interneurons) at 7–14 dpl, reduced *Unc13a* to 58%–79% (encoding Munc13-1) in a chronic manner, and reduced *Dgki* (encoding diacylglycerol-kinase Iota) to 55% at 14–28 dpl. Munc18-1 and Munc13-1 jointly control the quantal size of neurotransmitter vesicles in Diacylglycerol (DAG) dependence (Bhaskar et al., 2024), suggesting that presynapses may have altered neurotransmitter release. These reductions cannot be explained by neuronal loss, as they occur in specific time windows while most other presynaptic transcripts remain unaffected. This deficit is particularly interesting in light of electrophysiological data reporting synaptic strengthening of surviving synapses in the dentate gyrus at 3 days after ECL (Vlachos et al., 2012b; Vlachos et al., 2013). Reduction of presynaptic inhibition could contribute to synaptic upscaling (Wen and Turrigiano, 2024) *in vivo*.

Growth cone attraction efforts appeared to peak at 3–7 dpl. As one of the few factors upregulated in the OML and GCL after entorhinal denervation, the mainly interneuron-expressed *Nrp1* (encoding neuropilin-1, a co-receptor for semaphorin 3E and reelin) appears to represent a lesion-specific upstream regulator in a delayed transient and putatively compensatory manner (He and Tessier-Lavigne, 1997; Kohno et al., 2020).

#### Remodeling response of OML neuronal somata

Several factors with somatodendritic distribution were also downregulated. As a marker of granule cells (Muller et al., 2005), *Calb1* was reduced to 40% by 1 dpl and remained significantly deficient until 7 dpl. This observation mainly reflects changes in granule cell dendrites in the OML, but the denervation of CALB1-positive interneurons and semilunar granule cells (SGCs) might also contribute. As a marker of neuronal activity loss, early transient downregulations until 7 dpl affected *Sphkap*, which couples excitation to Ca^2+^-activated PKA signaling (Vierra et al., 2023), showing a maximal deficit at 1 dpl that recovered steadily. A contrasting acute and chronically sustained upregulation was observed for *Jun*, known for its role in the neural injury response following entorhinal denervation (Haas et al., 1993; Haas et al., 1999).

Denervation of interneurons was evident, as indicated by a chronic downregulation of *Gad1* to 54%–73%, an acute, prolonged downregulation of *Gabra1* to 50%, and a delayed reduction of *Pvalb* to 36%–47%. Overall, early on, the CALB1-positive granule cells and interneurons, and later on, also PV-positive interneurons, were strongly affected by dedifferentiation, in line with published reports (Nitsch and Frotscher, 1991; Nitsch et al., 1992; Nitsch, 1993).

#### Remodeling response of OML spines

The data identified *Neto1* as a selectively and strongly affected spine factor. The massive downregulation to 39%–59% for *Neto1* presumably impacted its function as a transmembrane factor containing a peptidase domain and an LDL receptor domain, as it acts as a potential SEMA3E/SEMA3F receptor (Gay et al., 2011; Mahadevan et al., 2021). This observation is consistent with the loss of 30%–40% of dendritic spines following ECL (Vuksic et al., 2011).

### II. Transcriptional adjustments occurring in the GCL

**Figure 4:**
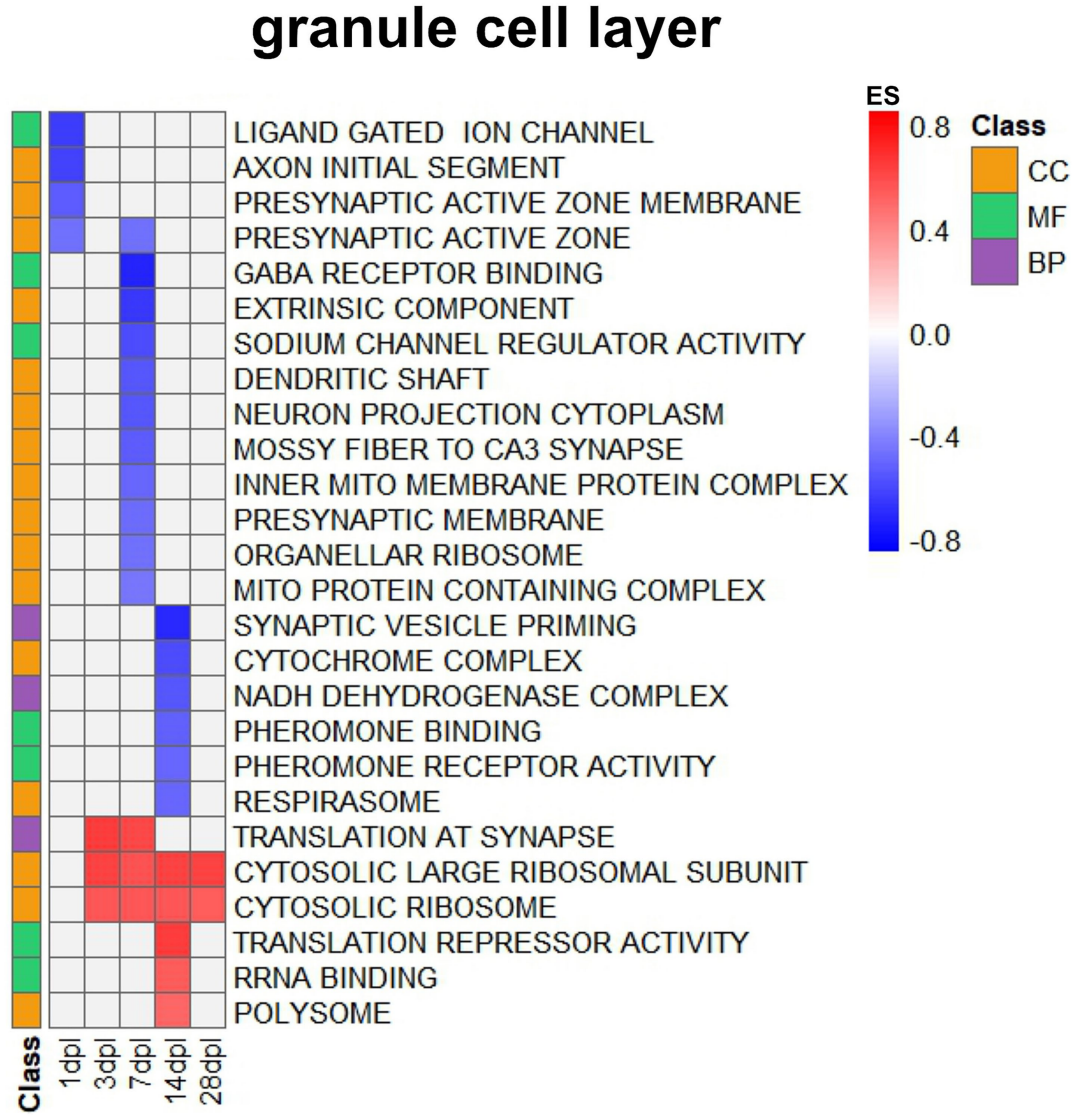
Pathway enrichment analysis via GSEA, showing GO terms in the dentate GCL after entorhinal lesion. Transcripts ranked by fold change were analyzed using Gene Set Enrichment Analysis (GSEA) software. The heatmap displays significantly enriched Gene Ontology (GO) terms in the granule cell layer at different time points after the lesion (dpl). Rows correspond to GO terms from the categories Cellular Component (CC, orange), Molecular Function (MF, green), and Biological Process (BP, violet), indicated by the color-coded class bar on the left. The color intensity of the heatmap reflects the enrichment score (ES), with red indicating positive and blue indicating negative enrichment.

At the systems biology level, GSEA software in the GCL (Supplementary Tables S5–S7) showed the following: The entorhinal denervation first triggered downregulations (Figure 4) at 1 dpl for axonal conductance (GOCC terms axon initial segment) and, in a sustained manner, for presynaptic and dendritic excitation (GOCC terms presynaptic active zone, presynaptic membrane, synaptic vesicle priming, dendritic shaft, neuron projection, mossy fiber to CA3 synapses) and mitochondrial activity (GOCC terms inner mitochondrial membrane protein complex, respirasome, cytochrome complex), sustained between 3 and 14 dpl. The impaired neural activity appeared to also include the mitochondrial translation machinery at the 7 dpl peak (GOCC term organellar ribosome). The reduction of signaling factors was evident at 1–7 dpl (GOMF terms ligand-gated ion channel, GABA receptor binding, sodium channel regulator activity, pheromone binding, and pheromone receptor activity). Again, at the 7 dpl peak, the impairment of synaptic transmission and respiratory complex-I activity became detectable (GOBP terms synaptic vesicle priming and NADH-dehydrogenase complex assembly).

The entorhinal denervation secondly triggered upregulations (Figure 4) of protein synthesis factors (GOCC terms for cytosolic ribosomes, cytosolic large ribosomal subunit, polysomes) from 3 dpl onwards. This was accompanied by an elevated demand for translation control factors and for rRNA binding molecules (GOMF terms) at the 7 dpl peak. As a presumptive repair effort, synaptic translation was found to increase at 3–7 dpl (GOBP terms). Thus, particularly in the GCL, a delayed activation of ribosomal translation was conspicuous, with synaptic protein synthesis peaking at 3–7 dpl. The apparent lack of consistency of GCL pathway enrichments across time in Figure 4, in contrast to OML enrichments in Figure 3, is probably due to the small sample size and low statistical power, given that many more individual dysregulations showed consistency over time in the GCL than in the OML according to Figure 2. These general pathway effects are expected in brain tissue undergoing reorganization, with our datasets now providing additional information on their temporal coordination.

Complementary enrichment studies across the transcriptomes via the STRING Heidelberg tool, regarding the pathways compiled in the KEGG and Reactome databases, again confirmed most effects (Supplementary File S2). In addition, significant activation of MAPK signaling cascades (presumably stress-induced) was detected at 1 and 14 dpl.

In analogy to OML findings of low semaphorin (*Sema3e*/*Sema5a*) and altered neuropilin (*Nrp1* up / *Neto1* down), prominent among GCL downregulations were the transient reduction of *Nrp2* versus induction of *Nrp1*, as well as decreased *Epha5*, *Epha7*, and *Epha6*, together with *Usp33*, as factors for axon growth and cell migration (Tear, 1998; Yuasa-Kawada et al., 2009). Also conspicuous regarding general neurotrophic signaling was the diminished expression of *Bdnf* ligand and *Erbb4* receptor, providing a credible explanation for the dedifferentiation of local neurons (Tables 3, 4).

**Table 3:** Downregulated transcripts in the hippocampal GCL after entorhinal lesion.

Gene symbol	Transcript ID	1 dpl *p*-value	1 dpl FC	3 dpl *p*-value	3 dpl FC	7 dpl *p*-value	7 dpl FC	14 dpl *p*-value	14 dpl FC	28 dpl *p*-value	28 dpl FC	HMP
Lrrtm3	10369752	0.01	0.37	0.04	0.39	0.06	0.42	0.08	0.46	0.09	0.47	0.03
Nrp2	10346843	0.02	0.65	0.03	0.61	0.05	0.64	0.07	0.67	0.10	0.71	0.04
Avpr1a	10366707	0.04	0.86	0.01	0.77	0.07	0.84	0.13	0.87	0.06	0.84	0.03
Atxn10	10425966	0.02	0.76	0.01	0.66	0.06	0.76	0.15	0.82	0.02	0.69	0.02
Usp33	10496919	0.01	0.74	0.01	0.65	0.01	0.68	0.06	0.78	0.06	0.77	0.01
Igf2r	10447799	0.02	0.61	0.03	0.56	0.04	0.57	0.06	0.62	0.05	0.61	0.03
Hlf	10389786	0.02	0.72	0.02	0.63	0.02	0.64	0.05	0.71	0.08	0.74	0.03
Egfr	10374366	0.02	0.76	0.05	0.75	0.05	0.75	0.05	0.76	0.17	0.83	0.04
Glra2	10607792	0.02	0.36	0.02	0.26	0.06	0.35	0.03	0.30	0.09	0.42	0.03
Mapk1	10433940	0.01	0.80	0.06	0.82	7.72E-04	0.64	0.01	0.76	0.05	0.82	3.30E-03
Calb1	10503416	8.97E-04	0.46	7.38E-03	0.48	0.01	0.51	5.41E-03	0.48	0.05	0.63	3.24E-03
Bdnf	10474399	9.53E-03	0.69	2.89E-03	0.55	1.77E-03	0.52	8.53E-04	0.50	2.61E-04	0.44	8.31E-04
Nfia	10506031	2.37E-04	0.39	3.33E-03	0.43	0.04	0.60	0.04	0.61	0.03	0.59	1.09E-03
Smarca2	10462237	1.47E-04	0.63	1.63E-03	0.65	1.34E-03	0.64	3.47E-03	0.69	2.11E-03	0.67	5.60E-04
Gtf2h5	10441489	3.03E-05	0.54	1.15E-04	0.50	1.40E-04	0.51	6.19E-04	0.59	4.14E-04	0.57	9.45E-05
Lin7b	10563253	1.90E-03	0.46	0.02	0.49	4.30E-03	0.41	0.02	0.50	0.03	0.54	0.01
Scn1a	10483299	2.88E-03	0.41	2.72E-04	0.19	4.21E-03	0.33	1.06E-03	0.27	2.40E-04	0.20	5.33E-04
Syt17	10567289	0.02	0.47	0.03	0.40	0.03	0.39	0.06	0.48	0.02	0.39	0.03
Epha5	10530870	1.62E-03	0.43	6.07E-03	0.41	4.26E-03	0.39	4.06E-03	0.40	1.47E-03	0.34	2.57E-03
Epha6	10440216	2.99E-03	0.28	0.05	0.40	0.03	0.36	0.05	0.40	0.06	0.42	0.01
Epha7	10503659	0.03	0.72	0.03	0.66	0.01	0.59	0.03	0.67	0.01	0.62	0.02
Chl1	10540298	3.78E-04	0.42	2.02E-03	0.41	3.07E-03	0.44	1.60E-03	0.41	1.20E-03	0.40	1.02E-03
Ntng1	10501468	8.69E-04	0.22	4.84E-04	0.11	4.24E-04	0.11	5.16E-03	0.23	1.31E-03	0.16	7.66E-04
Ank2	10501971	0.01	0.67	0.01	0.58	0.01	0.59	0.07	0.71	0.01	0.60	0.02
Lingo2	10512044	0.05	0.48	0.01	0.26	2.58E-03	0.17	0.05	0.39	0.09	0.46	0.01
Basp1	10427895	0.02	0.86	0.15	0.89	2.08E-03	0.73	0.04	0.84	0.04	0.85	0.01
Cnksr2	10607562	5.45E-04	0.80	1.79E-03	0.78	1.25E-04	0.70	3.19E-05	0.67	3.38E-05	0.67	7.01E-05
D0H4S114 / Nrep	10458046	3.06E-05	0.27	1.96E-04	0.26	5.09E-04	0.31	3.65E-04	0.30	3.81E-04	0.30	1.11E-04
Ano3	10485718	2.99E-04	0.32	1.02E-03	0.28	1.52E-03	0.30	1.19E-03	0.30	3.41E-04	0.24	5.71E-04
Rgs13	10358399	1.11E-04	0.06	1.34E-03	0.08	1.74E-04	0.03	4.24E-05	0.02	1.32E-05	0.01	4.36E-05
Gabra1	10385297	0.03	0.68	0.02	0.56	0.19	0.75	0.04	0.63	0.04	0.62	0.04
Mtap2 / Map2	10347036	0.03	0.81	0.05	0.79	0.10	0.83	0.03	0.77	0.02	0.75	0.03
Gabra2	10530406	1.55E-03	0.20	1.34E-03	0.11	0.02	0.25	2.80E-03	0.15	3.41E-03	0.16	2.39E-03
Adamts3	10531173	0.02	0.35	0.09	0.39	0.07	0.36	0.05	0.33	0.04	0.33	0.04
Ralgps2	10359201	2.04E-05	0.60	5.54E-04	0.64	1.08E-04	0.58	3.17E-05	0.54	5.24E-05	0.56	4.51E-05
Vps41	10403765	6.33E-05	0.48	6.37E-04	0.49	3.14E-04	0.46	7.68E-04	0.51	1.89E-04	0.44	1.84E-04
Lrrtm1	10539169	3.45E-03	0.36	9.46E-03	0.33	9.50E-03	0.33	7.21E-03	0.32	0.01	0.37	0.01
Arhgap12	10457250	5.52E-04	0.39	2.21E-03	0.36	0.02	0.51	0.01	0.48	0.03	0.55	2.05E-03
Grm7	10540509	1.26E-03	0.33	2.47E-03	0.27	1.35E-03	0.24	4.28E-03	0.31	1.94E-03	0.27	1.86E-03
Grik2	10368999	1.26E-03	0.53	7.55E-03	0.54	9.89E-03	0.56	0.01	0.58	0.03	0.64	4.36E-03
Cadm2	10440314	1.38E-04	0.47	1.28E-03	0.48	2.98E-03	0.53	1.00E-03	0.48	5.33E-04	0.45	4.44E-04
Adam11	10381619	1.44E-03	0.48	2.03E-03	0.39	1.21E-04	0.25	3.78E-04	0.32	1.19E-03	0.38	3.87E-04
Adam23	10346882	1.43E-03	0.29	9.70E-03	0.31	3.18E-03	0.24	1.46E-03	0.21	1.10E-03	0.19	1.85E-03
Dlg2	10554900	0.03	0.65	0.07	0.64	0.05	0.62	0.07	0.65	0.01	0.52	0.03
Lrrc7	10503054	0.01	0.77	0.22	0.86	0.05	0.78	0.05	0.78	0.06	0.79	0.04
Nrcam	10395553	4.10E-03	0.59	0.12	0.74	0.05	0.67	0.06	0.69	0.04	0.66	0.02
Hgf	10519857	0.28	0.91	0.05	0.77	0.03	0.75	0.01	0.69	0.01	0.70	0.02
Vsnl1	10399407	0.10	0.85	0.02	0.73	0.01	0.71	0.17	0.85	0.07	0.80	0.03
Mmp3	10583071	0.19	0.88	0.02	0.70	0.02	0.71	0.01	0.66	0.01	0.68	0.01
Snap47	10386388	0.10	0.66	0.05	0.50	0.04	0.49	0.06	0.53	0.03	0.48	0.05
Prss12	10495854	0.14	0.55	0.05	0.33	0.02	0.25	0.08	0.40	0.07	0.37	0.05
Stxbp5	10367775	0.41	0.81	0.04	0.45	0.04	0.46	0.70	0.88	0.33	0.72	0.09
Fjx1	10485402	0.57	0.90	0.02	0.51	0.01	0.46	0.13	0.67	0.06	0.60	0.03
Erbb4	10355278	0.05	0.62	0.02	0.45	0.03	0.47	0.50	0.82	0.38	0.77	0.05
Cabp1	10532956	0.16	0.76	0.26	0.76	0.04	0.57	0.05	0.60	0.23	0.75	0.08
Pcdhb16	10455108	0.13	0.66	0.06	0.50	0.04	0.46	0.02	0.43	0.03	0.45	0.04
Gabrb2	10375245	0.11	0.72	0.17	0.69	0.34	0.78	0.03	0.55	0.02	0.52	0.05

**Table 4:** Upregulated transcripts in hippocampal GCL after entorhinal lesion.

Gene symbol	Transcript ID	1 dpl *p*-value	1 dpl FC	3 dpl *p*-value	3 dpl FC	7 dpl *p*-value	7 dpl FC	14 dpl *p*-value	14 dpl FC	28 dpl *p*-value	28 dpl FC	HMP
Rab17	10356568	0.04	1.22	0.04	1.31	0.05	1.28	0.15	1.18	0.14	1.19	0.06
Pitx3	10468174	0.03	1.32	0.04	1.41	0.10	1.31	0.19	1.22	0.05	1.37	0.06
Tcfeb	10445729	0.03	1.23	0.03	1.31	0.01	1.40	0.08	1.23	0.24	1.14	0.03
Hcrt	10391273	0.04	1.27	0.02	1.49	0.03	1.40	0.17	1.21	0.09	1.28	0.04
Lmna	10499394	3.88E-06	3.48	2.73E-05	3.66	5.96E-05	3.24	4.51E-05	3.23	1.48E-05	3.86	1.25E-05
Rnf213	10383208	6.10E-10	4.79	1.54E-08	4.21	1.06E-08	4.49	8.31E-09	4.42	5.38E-09	4.77	2.38E-09
Cacng5	10392374	5.07E-04	3.58	4.74E-03	3.29	4.89E-03	3.27	3.09E-04	5.72	1.19E-03	4.20	7.74E-04
Lgi2	10530029	6.67E-05	4.38	0.02	2.23	0.02	2.25	0.01	2.29	0.02	2.21	3.28E-04
Stac	10597309	3.14E-05	1.80	3.78E-04	1.75	2.89E-04	1.79	4.67E-04	1.69	4.38E-04	1.69	1.18E-04
**Nup210l**	10,493,690	8.18E-08	19.39	8.71E-07	19.68	6.04E-07	22.44	3.09E-07	25.57	8.42E-07	17.87	2.57E-07
Setd1a	10557758	6.19E-04	2.09	1.52E-03	2.34	7.73E-03	1.91	5.21E-03	1.95	0.02	1.70	1.88E-03
Nov / Ccn3	10424119	1.16E-05	1.95	5.98E-05	2.05	9.51E-05	1.97	6.00E-05	2.00	1.67E-04	1.83	3.67E-05
Chrnb2	10499643	1.70E-03	3.05	1.94E-03	4.23	8.01E-03	3.10	4.59E-04	5.61	1.86E-03	4.05	1.27E-03
Fgf5	10523490	2.39E-03	2.04	1.25E-03	2.85	0.04	1.74	4.55E-03	2.27	4.30E-03	2.29	2.95E-03
Smn1	10406881	7.59E-03	3.39	6.03E-03	5.39	0.35	1.59	6.70E-03	4.91	0.03	3.31	0.01
Akap1	10389701	0.04	1.88	0.04	2.30	0.02	2.72	0.01	2.97	7.49E-03	3.23	0.02
Ablim3	10459262	0.01	3.58	0.11	2.67	0.09	2.93	0.03	4.22	0.02	4.81	0.03
Gpr68	10402136	3.95E-04	2.57	6.13E-03	2.25	0.02	1.91	3.45E-03	2.37	0.01	2.03	1.60E-03
Dlg4	10377725	0.02	1.32	0.05	1.34	0.23	1.18	0.02	1.39	0.03	1.38	0.03
Sorcs2	10529515	1.88E-03	1.96	5.20E-03	2.12	4.75E-04	2.98	6.20E-03	2.02	5.36E-03	2.06	1.57E-03
Sorcs3	10463875	6.45E-04	3.37	0.02	2.57	0.01	2.71	3.79E-03	3.24	6.21E-03	2.94	2.35E-03
Itsn2	10394288	0.05	1.42	0.13	1.40	0.04	1.63	0.03	1.65	0.04	1.60	0.04
Notch2	10494595	0.49	1.16	0.06	1.80	0.02	2.14	0.03	2.02	0.03	2.04	0.04
Ephb1	10596095	0.05	1.58	0.05	1.88	0.03	2.06	0.04	1.86	0.01	2.34	0.03
Mtcl1 /1110012J17Rik	10452538	0.22	1.18	0.01	1.67	0.03	1.57	0.01	1.71	0.01	1.70	0.02
Nrp1	10576639	0.15	1.42	0.03	2.15	0.01	2.40	0.02	2.30	0.01	2.79	0.01
Marcksl1	10508465	0.44	1.14	0.07	1.55	0.04	1.66	0.08	1.49	0.01	1.94	0.03
Tspyl2	10607346	0.07	1.50	0.06	1.76	0.05	1.83	0.03	1.94	0.04	1.88	0.04
Gem	10503334	0.06	1.48	0.02	1.95	0.04	1.83	0.05	1.71	0.11	1.52	0.04
Flrt3	10488147	0.26	1.14	0.15	1.25	0.01	1.56	0.04	1.38	0.21	1.20	0.04
Stim2	10521950	0.48	1.07	0.13	1.23	0.81	1.03	0.05	1.31	7.76E-03	1.50	0.03
Sip1 / Zeb2	10482448	0.06	1.70	0.09	1.88	0.05	2.08	0.04	2.13	0.02	2.56	0.04
Ptprn2	10399121	0.74	0.95	0.10	1.43	0.14	1.36	0.03	1.62	0.01	1.84	0.03

#### Remodeling response of glia in the GCL

A strong and chronic upregulation with a maximum at 14 dpl stood out for *Nup210l*, which is expressed preferentially in glial and vascular cells as well as neural progenitors (Orniacki et al., 2023), again suggesting that neural stem cell (NSC) differentiation is modulated. The induction of *Notch2* was significant in the late period of 7–28 dpl, while regeneration and differentiation were ongoing. NOTCH2 is selectively expressed in NSCs, and its signaling is known to maintain NSC quiescence while supporting radial glia (Engler et al., 2018).

Later upregulation at 14–28 dpl was documented for *Sip1* (encoding Zinc Finger E-Box Binding Homeobox 2, ZEB2) as a master regulator of astrogliosis (Vivinetto et al., 2020; Vivinetto and Cave, 2021). In sum, there is no evidence of increased glial mass in the GCL, but the datasets indicate a change in glial differentiation and effects on dentate gyrus stem cells.

#### Remodeling response of the GCL extracellular matrix (ECM)

In perfect alignment of OML and GCL, the levels of the reelin inactivation factor *Adamts3* were again downregulated. In the GCL, this occured only at the beginning and end of the observation period, with smaller effect sizes. This may influence the immediate reprogramming of stem cells to astrocytes or to newborn granule neurons, in cooperation with reelin and Notch signaling (Hashimoto-Torii et al., 2008), while later supporting their migration, neurite extension, and excitability. Transcriptional downregulation of three proteases (*Adamts3*, *Prss12, Mmp3*) and two NSC quiescence factors (*Notch2*, *Setd1a*) points to ECM remodeling in the GCL, although this region was not directly denervated by ECL.

#### Remodeling response of excitatory and inhibitory postsynapses and spines

Temporally specific deficits of spine adhesion factors were evident in the GCL. Among the downregulated genes, two glycosylated synaptic cell adhesion molecules, namely postsynaptic *Lrrtm3* and possibly presynaptic *Lrrtm1*, were altered, only at 1–3 dpl for *Lrrtm3*, but during 1–28 dpl for *Lrrtm1*.

Cellular efforts to compensate for the neurotrophic state were conspicuous and constitute potential targets for therapeutic interventions. The chronic inductions, peaking at 7 dpl for *Sorcs2* (postsynapse/neuropil distribution in RNA-seq) and at 1 dpl as well as 14 dpl for *Sorcs3* (soma distribution in RNA-seq), are noteworthy (Lane et al., 2012).

#### Remodeling of GCL dendrites

Downregulation at 3–7 dpl occurred for NOTCH-inducible *Fjx1*, which is a Golgi kinase, promoter of FAT3 signals, and inhibitor of dendrite extension (Deans et al., 2011). Chronic downregulation, with a minimum at 28 dpl, was observed for *Mtap2* (encoding microtubule associated protein 2). In line with previous data (Steward and Wallace, 1995), these factors appear to modulate dendrite trafficking. Conversely, upregulation at 1–3 dpl was found for interneuron-expressed *Rab17* (encoding Ras-Related Protein Rab-17), which mediates transcytosis, dendrite morphogenesis, and postsynaptic development (Hunziker and Peters, 1998; Mori et al., 2013).

#### Remodeling response of GCL neurites

Chronically decreased levels, with a minimum at 7 dpl, were found for *Ntng1*, a marker of mature granule cells and a secreted axon and dendrite guidance factor (Zhang et al., 2016; Hochgerner et al., 2018). Downregulation from 1 to 14 dpl was observed in the GCL, as previously seen in the OML, for *Lrrc7* (encoding densin-180). Interestingly, LRRC7 is located in dendrites and axon initial segments (Apperson et al., 1996). Downregulation at 7–14 dpl was documented for *Cabp1* (encoding caldendrin), which directly couples postsynaptic calcium signaling to actin remodeling in dendritic spines (Mikhaylova et al., 2018). Notably, caldendrin plays a role in the localization of the spine apparatus organelle (Konietzny et al., 2023), a specialized form of endoplasmic reticulum that is crucial for Hebbian and homeostatic synaptic plasticity in granule cells (Deller et al., 2003; Vlachos et al., 2013). Following entorhinal denervation, the spine apparatus is reorganized within the granule cell dendritic tree (Deller et al., 2006). The data are consistent with reduced neurotransmission during the first half of the observation period.

Compensatory changes were also identified: Upregulation from 7 dpl, with a maximum at 28 dpl, was prominently observed for *Marcksl1*. All upregulated factors in GCL neurites during the regeneration/differentiation stage are modulators of AMPA receptors and actin fibers.

#### Remodeling response of GCL somata

The chronically decreased levels, with a minimum at 1 dpl, for *Calb1* (encoding calbindin-1), a granule cell marker, suggest that their differentiation state is impaired. The most significant and substantial downregulation (with a minimum <1% at 28 dpl) pertained to the retrograde signaling factor *Rgs13*. The PKA-dependent RGS13 protein acts in the nucleus to inhibit CREB-mediated gene expression (Xie et al., 2008).

Upregulation between 7 and 14 dpl occurred for *Flrt3*, which is involved in neuronal migration, adhesion, and repulsion, mediates netrin-1 responsiveness, functions as a latrophilin ligand during spine development, and shows preferential expression in granule cells of the dentate gyrus (O’Sullivan et al., 2012).

Several membrane dynamics factors expressed in interneurons exhibited decreased levels. Downregulation at 3–7 dpl was observed for *Snap47* (encoding an interactor of syntaxin-17) and for *Stxbp5* (encoding syntaxin-1-binding protein 5, also known as Tomosyn). Additionally, membrane receptor transcripts showed decreased levels. Chronic downregulation, with a minimum at 3 dpl, was noted for *Gabra1* and *Gabra2*. Downregulation from 14 dpl, reaching a minimum at 28 dpl, occurred for *Gabrb2*, an extrasynaptic mediator of tonic GABAergic inhibition (Marchionni et al., 2007).

Conversely, upregulation from 7 to 28 dpl was observed for *Tspyl2*, a component of a transcriptional complex that interacts with CREB-binding protein, modulating the expression of neurotrophic factor *Bdnf* and NMDA glutamate receptors *Grin2a/b/c* in response to neuronal activity (Tsang et al., 2014; Liu et al., 2019). Regarding the amplification of neuronal signals, upregulation from 14 dpl, peaking at 28 dpl, was noted for *Stim2*, which acts as a NOTCH-dependent endoplasmic reticulum Ca2 + sensor to regulate neuronal calcium homeostasis, e.g., in presynapses, and also modulates glutamate receptor activity (Chanaday et al., 2021).

In relation to reelin signals from lost entorhinal afferents, upregulation from 3 dpl, with a maximum at 28 dpl, occurred for interneuron-expressed *Nrp1* (encoding neuropilin-1). The NRP1 protein specifically binds to C-terminal reelin, acting as a coreceptor for VLDLR. The interaction of the NRP1/VLDLR protein complex with reelin is essential for normal dendritic development (Kohno et al., 2020).

#### Remodeling response of the GCL axon initial segments

Following entorhinal denervation of the dentate gyrus, significant expression changes for AIS-associated factors that control efferent signaling were found in the GCL, with a majority being downregulated. Notably, several genes associated with the structural integrity of the AIS, e.g., *Nrcam* and *Ank2*, showed a decrease in their expression levels (with a minimum at 1 dpl and 3 dpl, respectively), suggesting early remodeling of the AIS. In addition, downregulations occurred for factors critical for the transport and clustering of specific channels to the AIS, including primarily the mainly interneuron-expressed *Scn1a*, which had minimal levels in the GCL at 7 dpl, and *Dlg2*, which had minimal levels in the GCL at 28 dpl. Furthermore, several transcripts for ion channels and receptors were also downregulated, e.g., *Gabra2*, which had minimal levels in the GCL at 3 dpl.

Conversely, upregulation from 3 dpl with a maximum at 14 dpl was observed for *Mtcl1*, which has been demonstrated to play an important role in the maintenance of the AIS in Purkinje cells (Satake et al., 2017). Thus, this gene may have a pivotal role in stabilizing the AIS and supporting recovery and a return to normal firing rates following the lesion (Reeves and Steward, 1988).

#### Remodeling response of the GCL axons and presynapses

As the earliest injury response relevant to GCL axons, *Usp33* showed downregulation until 7 dpl, with a minimum at 3 dpl. Chronic downregulation was observed for several axon guidance family members, but in successive stages: interneuron-expressed *Epha6* showed a minimum at 1 dpl, *Epha7* at 7 dpl, and *Epha5* at 28 dpl.

Chronic upregulations with maximal levels at 3 dpl during the repair stage were observed for two neurite modulators that have established main roles in axonogenesis: First and foremost, *Smn1* (encoding Survival Motor Neuron Protein, also known as Gemin 1) serves as a structural component in the SMN complex, playing a critical role in the outgrowth, differentiation, and survival of axons (McWhorter et al., 2003; Oprea et al., 2008). Second, with a maximum at 3 dpl, *Fgf5* is a mostly neuron-expressed trophic ligand with important roles during neurogenesis (Hughes et al., 1993; Reuss and Von Bohlen Halbach, 2003). Selective upregulation at 14–28 dpl, with a maximum at 28 dpl, was documented for interneuron-expressed *Ptprn2* (encoding a phosphatidylinositol phosphatase, which modulates secretory vesicle dynamics and localizes to synaptic boutons) (Jiang et al., 1998).

## Discussion

**Figure 5:**
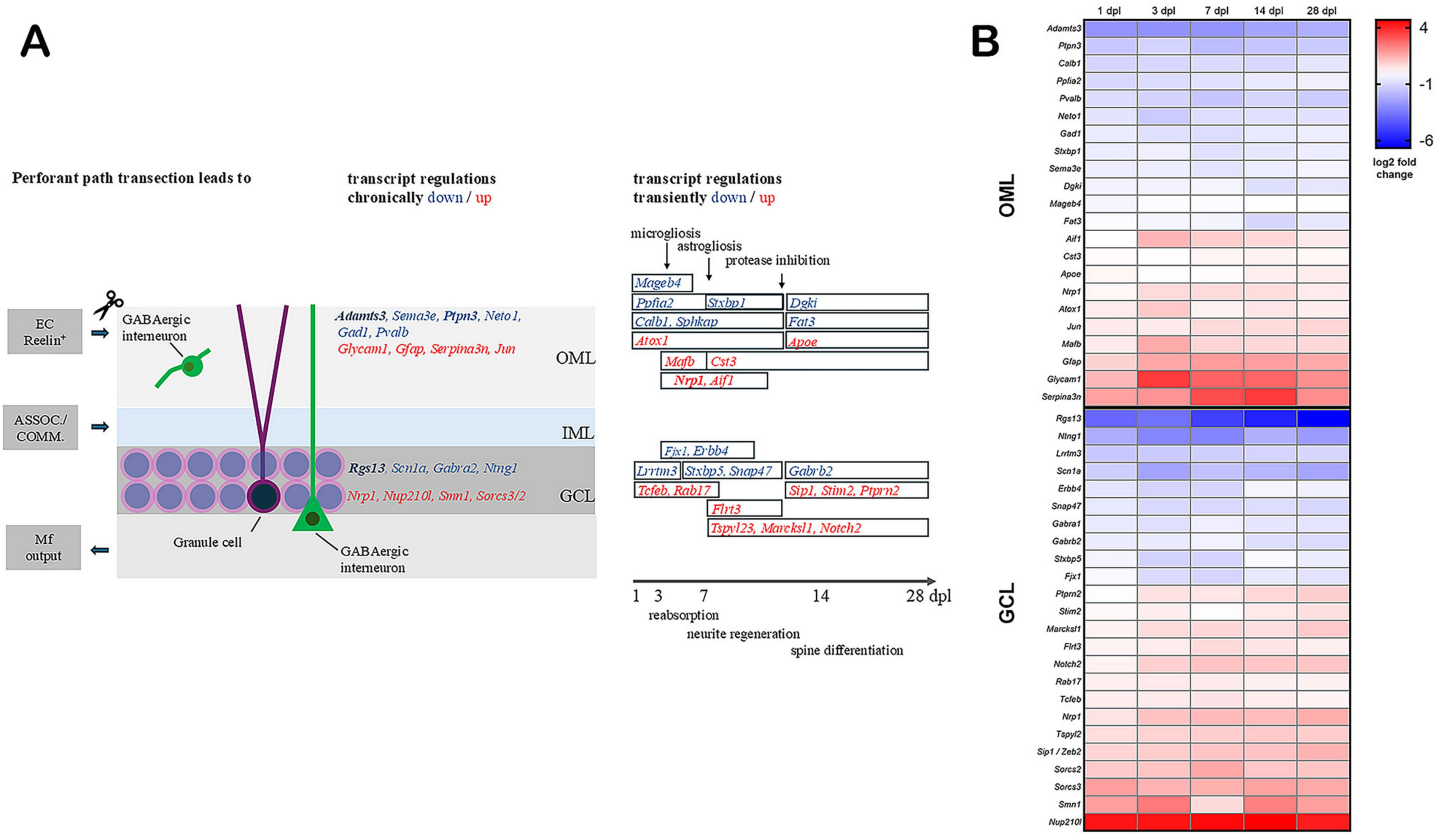
Synopsis of major detected, mostly neuronal gene expression changes in the dentate gyrus after entorhinal denervation. **(A)** On the left, a schematic image of the dentate gyrus outer molecular layer (OML), inner molecular layer (IML), and granule cell layer (GCL), their afferent input from the entorhinal cortex (EC) with reelin-positive projection neurons (surgically interrupted), from associational (ASSOC.) and commissural (COMM.) circuits, and their efferent output via mossy fibers (Mf), with prominent transcript dysregulation highlighted in bold letters. On the right, a depiction of the temporal dynamics, highlighting the peak of glial responses and the transition from protein breakdown to biosynthesis (above), compared to neural debris removal and differentiation stages (below), with supporting evidence from the transient regulation of relevant transcripts (in boxes delimiting the period of significant alteration, again with prominent factors in bold letters). The contributions of each transcript are detailed in the Discussion section. **(B)** Heatmap depicting log_2_ fold changes of factors from panel **A**. Downregulation is indicated in blue and upregulation in red. Log_2_ fold changes were color-mapped independent of statistical significance to visualize overall expression dynamics. Exact values are provided in Tables 1–4.

This work-intensive transcriptome survey in two dentate layers with five time points after entorhinal denervation represents a pioneering effort to define key genes showing increased or decreased expression for efficient tissue breakdown, compensatory transitory synaptic scaling, and subsequent circuit regeneration. A graphical summary (Figure 5) illustrates the main findings of our study.

### Global analyses of pathway enrichments with consistency over time

The unbiased detection of enriched GO terms for cellular components, molecular functions, and biological processes via GSEA analyses, as well as enriched pathways in the KEGG and Reactome lists via STRING analyses, showed consistent patterns across this time course. This agreement is remarkable, given that GSEA uses all transcripts together with their fold changes as input but ignores significance, while STRING uses only the transcripts with nominally significant dysregulations but ignores fold changes. Overall, early downregulations of neuroactive ligands, later downregulation of mainly glutamatergic neurotransmission factors, and late downregulations of respiratory factors, in parallel with early upregulations of debris degradation factors, subsequent upregulation of glial and inflammation factors, and late upregulation of ribosomal translation were documented by both approaches. These global effects are compatible with an injury response in three overlapping phases: (i) acute detachment of damaged fibers from their circuitry context, leading to subsequent signaling and trophic deficits; (ii) subsequent debris elimination together with microglial/immune and astroglial activation; (iii) late decreases in energy production, in parallel with protein synthesis efforts to achieve synaptic remodeling and network reconstruction.

### Early transcriptional changes following denervation and tissue breakdown

Following entorhinal denervation, we observed a reduction of presynaptic molecular markers, such as *Ppfia2* / *Unc13a* / *Grm7* to 48% / 58% / 65%, respectively, a reduction of the axon repellent *Sema3e* (Mata et al., 2018) to 60%, and a reduction of OML axon markers like *Nefl* / *Nefh* to around 70%. The reduction of these markers was, however, less than expected, as ~80% of synapses are lost following entorhinal denervation (Matthews et al., 1976), suggesting that other axons and presynapses, e.g., those from local interneurons (Deller et al., 1995) or CA3 pyramidal neurons (Drojdahl et al., 2002; Hainmueller and Bartos, 2020), remain in the denervated zone. Thus, these markers do not necessarily reflect the perforant path loss. Instead, they may serve as indicators of afferent tissue breakdown occurring in this region after denervation (Matthews et al., 1976; Caceres et al., 1983; Steward, 1994). Postsynaptic degradation may also underlie the delayed reduction to 47% for the AMPA-receptor modulator *Cnih2*, to 39% for the NMDA/kainate receptor modulator *Neto1*, and to 56% for the MGLUR effector *Homer2*, as spine components, consistent with the observed denervation-induced synapse and spine loss (Caceres et al., 1983; Vuksic et al., 2011). The immediately diminished levels of *Calb1* (in OML to 40% and in GCL to 46%), a marker of granule cell dendrite arborization (Yang et al., 2015), were recovering over time, reflecting the regeneration time course after denervation. Furthermore, the delayed drop to 36% for *Pvalb* (as a marker of PV + interneurons) and the immediate chronic drop to 54% for *Gad1* (as a GABA biosynthesis enzyme in inhibitory interneurons) mirror the denervation and subsequent dedifferentiation of local inhibitory interneurons (Nitsch and Frotscher, 1991; Nitsch et al., 1992; Nitsch, 1993).

### Prominent and lasting changes in reelin signaling pathways

An important finding of our study was the strong decrease to 14% for GC-expressed *Adamts3*, which encodes a degradation enzyme for reelin (Ogino et al., 2017). The reduction of *Adamts3* constitutes the strongest downregulation effect in the denervated molecular layer and may represent a compensatory effort to amplify the remaining excitatory signals (Pujadas et al., 2010). Notably, this downregulation was also observed in the GCL, where *Adamts3* nuclear transcription occurs. Reelin is present in layer II neurons in the entorhinal cortex that project to the dentate gyrus (Kobro-Flatmoen et al., 2016; Hamad et al., 2024) and in OML Cajal–Retzius cells (Del Rio et al., 1997). It plays a role in the lamination of the dentate gyrus and in GC positioning (Deller et al., 1999). Following transection of the perforant pathway in postnatal hippocampal slices, entorhinal axons require reelin to regenerate into the dentate gyrus (Wu et al., 2008). Furthermore, reelin potentiates glutamatergic excitation via enhanced tyrosine phosphorylation of NMDA receptors (Chen et al., 2005). The massive loss of reelin-expressing entorhinal axons following entorhinal denervation may also explain the strong OML downregulation to 30% for the GC-expressed tyrosine protein phosphatase *Ptpn3* (in trough shape with a minimum at 7 dpl), as a putative intracellular effector suppressing these growth signals (Laczmanska and Sasiadek, 2011; Tang et al., 2022). The strong OML downregulation to 39% of the interneuron spine component *Neto1*, a homolog of the SEMA3E/VLDLR receptor effector and reelin interactor NRP1 (Kohno et al., 2020), can also be interpreted in this context. Thus, important reelin-cleaving and -inhibiting enzymes and downstream signaling pathways are reduced following the lesion.

The most prominent transcriptional upregulation by the deprived neurons jointly in OML and GCL, albeit with a delay, was the increase of progenitor/interneuron-expressed (Andrews et al., 2017) *Nrp1* to 303% and 692%, respectively. The encoded NRP1 protein serves as a co-receptor for the secreted SEMA3E ligand, which is sensed by the PLEXIND1 receptor in entorhinal afferents (Chauvet et al., 2007). NRP1 also acts as a co-receptor for VLDLR (very low-density lipoprotein receptor) and specifically binds to the C-terminal region of reelin, an interaction shown to be essential for normal dendritic development in superficial layer neurons of the neocortex (Kohno et al., 2020). These findings are highly consistent between both dentate layers, and their exceptional effect sizes underline the well-established essential role of the extracellular matrix cue reelin for entorhinal axon guidance (Del Rio et al., 1997).

Whereas previous experiments had searched for reelin transcript induction after perforant path transection (Haas et al., 2000), our array data analysis raises the intriguing possibility that immediate and chronic reelin activation following denervation is not achieved by an increase in reelin expression. Rather, the downregulation of its repressor *Adamts3* and its putative intracellular effectors *Ptpn3* and *Neto1*, together with the delayed upregulation of its co-receptor *Nrp1*, might be the relevant downstream responses to the loss of reelin-expressing afferents.

### Evidence for the activation of compensatory pathways: neurotrophic signaling and synaptic upscaling

Neuronal efforts to compensate for diminished neurotrophic signals (GCL deficits occurred in an acute transient manner for *Avpr1a*, *Igf2r*, *Egfr*, *Hlf*, and sustainedly for *Bdnf*) during regeneration are probably best documented by GCL upregulation of postsynaptic-dendritic *Sorcs2* and mainly interneuron-expressed somato-axonal *Sorcs3* as promiscuous growth factor co-receptors, whose neuroprotective functions guard against neurodegenerative processes, e.g., in Alzheimer’s disease (Reitz et al., 2013; Malik and Willnow, 2020).

Compensatory putative upscaling of excitability was observed in the OML via strongly reduced *Vamp1* as a presynaptic vesicle release factor in GABAergic interneurons (Vuong et al., 2018), which aligns with a robust denervation of GABAergic basket neurons (Nitsch and Frotscher, 1991; Nitsch et al., 1992; Nitsch, 1993). Apart from this major effect, minor transient downregulations of synaptic vesicle priming/filling involved first *Ppfia2* at 1–14 dpl, then *Stxbp1* at 7–14 dpl, and finally *Dgki* at 14–28 dpl. In the GCL, we observed an extraordinary downregulation of *Rgs13* as a repressor of the constitutive transcription factor CREB (Xie et al., 2008), as well as upregulation of the inducible transcription factor and injury rescue mediator *Jun* (Herdegen and Leah, 1998). This was accompanied by a massive upregulation of *Cacng5* as a stargazin-related AMPA receptor modulator that increases peak currents (Kato et al., 2008) and a moderate upregulation of calcium homeostasis factors (*Gem*/KIR, *Stac*), which will also amplify excitatory signals. Increased levels of *Chrnb2* encoding a cholinergic sensor, *Lgi2* encoding a synaptic adhesion factor, and *Smn1* / *Fgf5* encoding axonal growth mediators may represent further efforts to strengthen the signal circuits. These findings are compatible with the post-lesional upscaling of surviving postsynapses, which has been demonstrated after entorhinal denervation *in vitro* (Vlachos et al., 2012b; Vlachos et al., 2013).

### Evidence for a reduced neuronal output following denervation

A deficient output from granule cells and interneurons is likely, given the massively decreased levels of *Ntng1* and mildly decreased levels of *Calb1*, *Gabra1*, *Pvalb*, and *Gad1* as markers of neuronal differentiation maturity (Raol et al., 2006; Hochgerner et al., 2018), as well as reduced levels of *Scn1a* and *Gabra2* as signal transduction factors in the axon initial segment (AIS) (Hedrich et al., 2014; Muir and Kittler, 2014; Hong et al., 2020), along with *Lrrc7* in the OML and GCL with AIS presence (Apperson et al., 1996) in addition to its established membrane-associated postsynaptic density role. Compensatory efforts at the AIS include the delayed upregulation of *Mtcl1* as a microtubule crosslinking factor (Satake et al., 2017). The reduction of AIS signal transduction factors is consistent with electrophysiological data showing a strong but transient reduction in granule cell action potential generation (Reeves and Steward, 1988). This decrease in granule cell activity may be even more pronounced in mice following denervation, given that these animals lack the crossed entorhinal pathway, which sprouts and reinnervates the molecular layer in rats (Deller et al., 2007).

### Reorganization of extracellular matrix, dendrites, and spines following denervation

The above chronic stable dysregulations must particularly take into account that neuronal tissue loss of around 30% and microglial proliferation act as confounders that may underlie transcript changes of average strength. These confounding effects will vary over time. Therefore, distinguishing between true down/up-regulations and tissue composition effects with a constant cutoff value is impossible, and only massive or extraordinary effect sizes merit detailed discussion.

However, among the dysregulations with a selective time window (or marked peak/trough shape), even minor fold changes deserve attention (see (Figure 5). During the initial lesion and tissue reabsorption stage, the GCL deficit of *Lrrtm3* reflects a temporal alteration in the density of spines receiving medial entorhinal afferents and signaling to mossy fibers after surgery (Kim et al., 2022). The immediate OML downregulation of *Mageb4* occurs when germ cells enter meiosis (Osterlund et al., 2000). The transient OML inductions of *Atox1* as a copper removal factor (Zhao et al., 2024), together with GCL inductions of *Rab17* as a membrane dynamics mediator (Mori and Fukuda, 2015) and *Tcfeb* as a transcription factor that controls the autophago-lysosomal pathway (Settembre et al., 2011), reflect the prominence of breakdown and removal factors. Upregulation of the protease inhibitor *Cst3* after 7 dpl (Bengtsson et al., 2008) appears to terminate ECM breakdown. Indeed, the ECM is profoundly reorganized following entorhinal denervation (Deller et al., 2000), and some of the above transcriptional changes may be linked to ECM remodeling after denervation.

Subsequent regenerative efforts extend beyond the upregulation of *Nrp1* from 3 dpl, which converts axonal repulsion into attraction signals (Chauvet et al., 2007). The promotion of dendrite branching and axonal sprouting might be reflected by the delayed transient downregulations of GCL *Erbb4* and *Fjx1* at 3–7 dpl, *Stxbp5* with *Snap47* at 3–7 dpl, and of OML *Fat3* subsequently at 14–28 dpl. The OML upregulations of *Stxbp1* govern dendritic arborization (Shen et al., 2020). Temporary GCL decreases of *Snap47* control axon branching (Shimojo et al., 2015). *Erbb4* promotes inhibitory connections (Luo et al., 2021). *Fjx1* inhibits dendritic growth as a Golgi kinase and promotes FAT3 signals (Probst et al., 2007). *Fat3* encodes a repressor of neuron migration, axon fasciculation, and dendrite development. *Fat3* mutation causes the formation of excess neurites (Aviles et al., 2022). Therefore, its late downregulation may reflect the need to minimize neurite retraction and unipolarity at this stage, as denervated dendrites start to recover and exhibit their limited re-growth (Vuksic et al., 2011) to achieve homeostasis (Platschek et al., 2016; Cuntz et al., 2021).

During the later regenerative stages, recovery of dendritic spine densities (Vuksic et al., 2011) may be linked to delayed GCL upregulations of *Tspyl2* as a nuclear CREB effector mediating the expression of several glutamate receptors, and of *Bdnf* as a neurotrophic factor (Liu et al., 2019), *Flrt3* as a mediator of glutamatergic synapse development and a determinant of spine number (O’Sullivan et al., 2012), *Marcksl1* as an actin stability and spine promoter (Tanaka et al., 2018), and *Notch2* as a repressor of neurogenesis (Engler et al., 2018). Spine stabilization may be enhanced by *Stim2* as a Ca^2+^ sensor of immature spines (Kushnireva et al., 2020). Indeed, increased spine stability during the regeneration phase has been reported to contribute to spine density recovery after entorhinal denervation *in vitro* (Vlachos et al., 2012a).

### Glial transcription indicates glial repair activity in the denervated zone

Microglia as well as astroglia show rapid and long-lasting responses to entorhinal denervation in mice (Schlaudraff et al., 2022). As novel glial correlates of OML repair activity, this transcriptome study identifies the following factors: firstly, *Glycam1* that stimulates extravasal infiltration by leukocytes (Imai et al., 1993), with increased expression levels peaking at 3 dpl, coinciding with the induction of the macrophage infiltration factor *Mafb*, and with peak levels of microgliosis markers *Aif1* and *Tyrobp* (Figure 1); secondly, the *Cxcl5* chemokine and *Ccr5* chemokine receptor levels that promote gliosis (Friedman-Levi et al., 2021; Cao et al., 2023) with maximum expression at 7 dpl, together with astrogliosis markers *Gfap* and *Vim*; thirdly, the protease inhibitor *Serpina3n* that controls gliosis (Zamanian et al., 2012), with maximum expression at 14 dpl, when the protease inhibitor *Cst3* also shows a mild peak. These OML data contrast with GCL findings, where most markers of reactive microgliosis/astrogliosis were unchanged, but *Nup210l,* a nuclear envelope factor (Orniacki et al., 2023), whose expression is mainly regulated in glia and progenitors, showed massive GCL upregulation that peaked at 14 dpl. Quite stable but strong GCL inductions were observed for mainly glia-expressed *Lmna* and *Rnf213*.

### Methodological limitations

The biggest advantage of microarrays is their global overview, so the factors mentioned above are truly prominent. While newer RNA-seq techniques produce solid data on the few strongly expressed genes, they provide scarce or no data on poorly expressed genes, whereas the well-established microarrays detect all known genes by at least one probe. Even for barely expressed transcription factors, the signal changes can be analyzed in a linear fashion without saturation artifacts. Thus, our approach enabled the identification and comparative ranking (by fold change) of numerous factors whose role in hippocampal lesion response was unknown or only reported in candidate-driven studies. Furthermore, it was now possible to understand the temporal order of their relative contributions within the 4 weeks after the lesion. This molecular panorama can serve as a basis for further investigations, asking which molecular switches are crucial for the remodeling and functional recovery of a denervated brain region.

Although microarrays provide an overview, they reveal only some aspects of the many molecular events occurring after entorhinal denervation. Firstly, the regulation of global transcripts constitutes only an initial activation of molecular programs that are subsequently modified and regulated. Alternative splicing, microRNAs, stimulus-dependent local mRNA translation, post-translational modification, protein trafficking, liquid–liquid phase separation, proteolytic cleavage and degradation, as well as membrane dynamics need to be considered to understand the complex spatial and temporal changes following denervation. Secondly, the analysis presented here is narrowly focused on a subset of ~20,000 genes active in mammalian cells. Non-coding, anonymous, poorly studied, or controversial RNAs were disregarded; thus, in the absence of any microRNA data or insight into translation regulation, it cannot be assumed that the corresponding protein levels have changed or that functional consequences ensue. We focused on neuronally expressed rather than glial expressed factors and paid special attention to transcripts with potential roles in neuronal development, plasticity, homeostasis, and regeneration. The manual selection of neuronal factors based on pre-existing insights into their roles in the hippocampus may have introduced a bias and reduced the comprehensiveness of our survey. Furthermore, future studies will be needed to further elucidate, e.g., the glial contribution to ECM remodeling, the microglial contribution to debris removal and synaptic plasticity, or the astroglial contribution to metabolic adaptations, ion/solute-dependent excitability, and differentiation. Thirdly, our data are preliminary because experimental validation cannot be performed for so many players. The fold changes of neuronal transcripts are usually too small and easily diluted, precluding subsequent validation efforts by immunoblots or immunocytochemistry. Therefore, functional analyses such as electrophysiology will be required to assess our transcriptome evidence. Nevertheless, to maximize the intrinsic validity of our dataset, we ensured that transcripts (i) reflected the lesion and its known consequences (e.g., *Sema3e* deficit as a marker of entorhinal axon loss (Mata et al., 2018), *Calb1* deficit as a marker of granule cell damage (Muller et al., 2005), *Gfap* and *Aif1* upregulation as markers of astrogliosis (Eng and Ghirnikar, 1994; Schlaudraff et al., 2022) and microgliosis (Ito et al., 1998), respectively), (ii) showed significant dysregulation in the same direction at several neighboring time points while not showing converse regulations for other time points or for other probes representing the same gene, and (iii) possibly exhibited parallel co-regulation for several components in the same pathway, perhaps during a restricted period.

This study is also limited in statistical power, given that only three animals were used per time point for the control or operated group. Therefore, subtle or variable effects may have escaped detection. This is particularly true at 7 dpl, where one animal had to be excluded from the analysis due to technical problems, and any findings have restricted robustness. This time point coincides with a maximum number of dysregulations, and even more impacts could have been detected there with a higher number of animals.

Thus, although our report is the first comprehensive spatial and temporal pattern analysis of transcriptional changes in a denervated brain region, it is merely a preliminary description of a complex multi-dimensional chain of events. The datasets we provide have revealed initial evidence for regulatory pathways that have not yet been implicated in brain reorganization. They represent a pioneering step toward formulating new hypotheses that can now be tested and verified (or falsified) using complementary approaches. Follow-up studies based on our work may ultimately lead to a deeper understanding of denervation-induced brain reorganization in the coming years.

## Data Availability

All original microarray data were deposited publicly at the Gene Expression Omnibus (GEO) depository, with the accession numbers GSE284333 (GCL) and GSE284334 (OML).
