# Supplementary material for: Spatiotemporal patterns of gene expression changes in the mouse dentate gyrus following entorhinal denervation
Source: Front Mol Neurosci. 2026 May 19;19:1758390. doi: 10.3389/fnmol.2026.1758390 (PMC13226496; doi:10.3389/fnmol.2026.1758390)
Supplement: Supplementary file 10 [file Data_sheet_1.docx]

**Results**

The analysis of expression changes across individual time points showed rapid responses already at 1 dpl, with a peak in the number of dysregulations at 3 dpl in the OML and at 7 dpl in the GCL. The number of transcript adjustments was reduced already to half at 28 dpl in the OML but was still ongoing at 28 dpl in the GCL. A prominence of upregulations was present in the OML, which mostly reflected gliosis (Fig. 2). The fold-change (FC) of expression regulations was smaller at 1 dpl in both regions, maximal for upregulations at 3 dpl in the OML (mainly macrophage and microglia factors), but otherwise quite constant afterwards in OML and GCL (Fig. 2). Venn diagrams in the OML showed only some 5% of all dysregulated transcripts to be significant with consistency for each time-point, versus 25% in the GCL (Fig. 2), suggesting more dynamics in OML transcription programs over time (likely reflecting repair), while GCL stress responses had a more uniform pattern (as permanent adaptations).

**I. Transcriptional adjustments occurring in the OML**

GSEA filtering of OML data emphasized that entorhinal deafferentation first triggered downregulations (Fig. 3) from 3 dpl onwards for the neurotransmission machinery (GOCC terms for postsynaptic density, main axon, presynaptic active zone) and neuronal respiration (GOCC terms for cytochrome complex, respiratory chain complex IV, respirasome, exemplified in Table 1 by *Cox6a1* and *Cox1* decreases), in acute and sustained manner. Structural constituents of synapses (GOMF terms) were in diminished demand during the 7-14 dpl peak. Processes of synaptic trafficking and electron transport chain (GOBP terms) appeared impaired since 3 dpl.

The entorhinal denervation secondly triggered upregulations of protein/RNA/lipid degradation efforts (GOCC terms of proteasome, phagocytosis, autophagosomes, endoplasmic reticulum-associated degradation, vacuolar lysosomes), in acute and sustained manner. These efforts are performed mostly by microglia, with the help of additional invading macrophages. They occurred in parallel to the GOMF term for the S100 family, which represent well-known glial markers of damage in the nervous system. GOBP terms illustrated that the activation of glia and leukocytes was accompanied by the generation of superoxides (presumably during oxidative bursts of macrophages and microglia while they engulf toxic debris), and by elevated antigen presentation (presumably to assess the presence of foreign bodies or microorganisms among the breakdown products). These findings, which indicate reactive changes of various cell types (in particular glia), the activation of degradation and repair programs, altered neuronal signaling and overall energy consumption, are expected and appear in line with current concepts of brain repair.

In the following paragraphs with subtitles, a stratification within this “big data” landscape was attempted, subgrouping the transcript changes according to the neuronal compartments, temporal co-regulations and functional pathways, to maximize the recognition of co-regulation.

Manual selection of significantly changed mRNAs was done, adhering to the following criteria: (i) intrinsic validation upon significant effect reproduction at an adjacent time point; (ii) FC above 1.15 or below 0.87, for maximized pathway recognition; (iii) published roles for the shaping of neuronal subcompartments, if possible, in hippocampal studies. This focus on neuronal morphology changes, relating them to underlying waves of transcription programs, cannot be exhaustive within one manuscript. It will ignore short transient transcription patterns and anonymous mRNAs, as well as important aspects of glial contribution, excitability and metabolism, particularly by neglecting ion and solute trafficking factors.

The compilation of selected transcripts in the main Tables 1-4 used different color hues (red for upregulations, blue for downregulations) within a heatmap representation of FC, and nominal significances with p-values (in orange color hues), per factor and time point. This illustration allows readers to rapidly survey which differential regulations are prominent against a background of reductions where neuronal tissue loss and dedifferentiation act as confounders, and a background of inductions where microgliosis and astrogliosis may contribute. To enable relative comparisons to neuronal tissue loss and to gliosis gain, compartmental markers were included, firstly for axon markers such as *Sema3e/Nefl/Nefm/Nefh* (Table 1), secondly for astrocytes such as *Gfap/Vim*, thirdly for microglia such as *Aif1/Tyrobp* (Table 2). To visualize temporal co-regulations, factors are grouped first by acute transient dysregulations, then chronic changes, and finally delayed and terminal responses. Within such clusters, factors were ordered by their relevance for overall reprogramming of postsynapse – dendrite – soma – axon – presynapse. Columns from left to right indicate for each factor the gene symbol and transcript identity number within the microarray, for each time point the p-value and the FC of expression adjustment. Factors with prominent and consistent effect sizes in FC were highlighted in bold. To improve readability of the text, literature for each factor was listed in the Supplemental Tables as PubMed-ID numbers.

Immediate effects of entorhinal afferent loss in the shrinking OML tissue (Table 1) were exemplified by significant reductions at 1-14 dpl, with a minimum around 60% at 7 dpl, and normalization by 28 dpl, for Sema3e mRNA (encoding secreted ligand Semaphorin 3E) as their axon repellent. Similarly, a reduction at 1-7 dpl, to about 80%, for Sema5a (encoding transmembrane ligand Semaphorin 5A) as granule cell dendritic spine biogenesis factor was documented. Thus, an immediate marked affection of axon guidance cues was prominent.

The converse proportional gain of glia in OML tissue (Table 2) was reflected e.g. by significant chronic inductions of two cytoskeletal intermediate filaments, *Gfap* (1.8-3.5-fold) and *Vim* (1.4-2.2-fold) as astrocyte markers with maximum at 7 dpl, as well as two microglia markers with maximum at 3 dpl, the transmembrane factor *Tyrobp* (a protein tyrosine kinase and integrin binding phagocytosis activator, 1.3-2.2-fold), and *Aif1* (a cytokine/calcium-activated actin-polymerization / membrane ruffling / phagocytosis factor, 1.6-2.6-fold). Tables 2 and 4 were selected to include meaningful neuronal upregulations rather than gliosis factors.

In the search for interesting patterns or relevant individual regulators, we highlight affected intercellular signaling pathways beyond the well-studied glutamate-GABA-glycine-acetylcholine signaling: The acute and chronic OML deficit of *Nps* (encoding Neuropeptide-S) as anxiolysis and arousal mediating signal in the entorhinal and dentate projections provides one example in Table 1. Subsequent Tables also show GCL upregulation at 1-7 dpl for *Hcrt* (encoding the Narcolepsy-associated neuropeptide precursor PrePro-Hypocretin/Orexin), which conveys appetite and sleep signals from the hypothalamus to the hippocampus, providing another example.

An interesting pattern involved the GCL induction of *Tcfeb* (encoding the Transcription Factor EB, aka BHLHE35), which controls the expression of lysosomal biogenesis and autophagy factors, in the initial period 1-7 dpl when debris has to be removed. Subsequently in the late period 7-28 dpl, the OML upregulation of *Cst3* (encoding the cysteine protease inhibitor Cystatin C) suggests that debris breakdown is terminated, while simultaneous decreases of *Eef1a2* (encoding Eukaryotic Translation Elongation Factor 1 Alpha 2), which serves the enzymatic delivery of aminoacyl tRNAs to the ribosome, suggest that protein biosynthesis is still limited.

Also interestingly, the acute transient OML downregulation at 1-3 dpl of *Mageb4* (encoding Melanoma-Associated Antigen B4) as cytoplasmic membrane-associated, F-actin-filopodia overlapping factor with exclusive expression in non-proliferative stem cells, may suggest a neurogenesis response already at the initial stage. Lateron at 7-28 dpl, the induction of *Notch2* (encoding Neurogenic Locus Notch Homolog Protein 2, a membrane receptor) as neurogenesis repressor suggests that proliferation is finished and differentiation has started.

**Remodeling response of OML glia activators and repressors**

Specific glia factors were crucial for reabsorption of damaged tissue. During peak repair at 3-14 dpl, macrophage/glia-expressed Atox1 (encoding Antioxidant 1 Copper Chaperone, aka HAH1) was elevated with maximum at 3 dpl, as a factor responsible for copper removal from debris, whereas a chronic increase again with peak at 3 dpl was documented for *Ftl1* (encoding Ferritin Light Chain 1) as microglial factor for the homeostasis of iron. Taking into account the time-course of micro-/astrogliosis markers mentioned above, the chronic upregulation with maximum at 3 dpl for *Sparc* (encoding Secreted Protein Acidic And Rich In Cysteine, aka Osteonectin), as known entorhinal denervation-induced vascular and reactive astrocyte factor that interacts with ECM, cytokines and growth factors to modulate glutamate homeostasis (Liu et al., 2005), appeared to contribute to macrophage invasion. An induction at 3 dpl that was sustained until 14 dpl was documented for mainly microglia-expressed *B2m* (encoding Beta-2-Microglobulin), as secreted factor that is found on the surface of nucleated cells and mediates antibacterial functions, thus serving neuroinflammatory roles. It is also known to repress glutamatergic neurotransmission and neurogenesis. This microglial activation was possibly governed by *Mafb* (encoding MAFB/Kreisler Basic Region/Leucine Zipper Transcription Factor, aka KRML), as factor responsible for macrophage infiltration and adult microglia colonization after brain damage, as well as interneuron excitability, which exhibited delayed induction with maximum at dpl 3 and sustained upregulation until 28 dpl.

The subsequent activation of astrocytes around 7 dpl coincided with maximal activation of specific molecules. A delayed induction with maximum at 7 dpl was documented for neuron-inducible *Ccr5* (encoding C-C Motif Chemokine Receptor 5) as chemokine receptor that serves the communication between neurons and glia to promote astrogliosis. Similarly delayed upregulation with maximum at 7 dpl for *Cxcl5* (encoding C-X-C Motif Chemokine 5, aka ENA-78), as astroglial chemokine ligand that modulates synapses, represents its putative receptor ligand. Delayed upregulation with maximum at 7 dpl for *Gsn* (encoding Gelsolin) is known to occur in astrocytes after entorhinal deafferentation (Dong et al., 2006). Gelsolin is responsible for the capping of actin-multimerization and the motility of cell processes.

After tissue breakdown, the re-establishment of neurotransmission and neurotrophic mechanisms was modulated by specific glia factors. The prominent novel finding was the strong and chronic upregulation with maximal levels at 14 dpl for *Serpina3n* mRNA (encoding Serine Or Cysteine Proteinase Inhibitor Clade A Member 3, aka Alpha-1-Antichymotrypsin), as serine peptidase inhibitor that is produced by neurons and glia cells to control gliosis via the NFkB signaling pathway. Chronic upregulation with maximum at 14 dpl was also observed for *S100a6* (encoding S100 Calcium-Binding Protein A6, aka Calcyclin) as astrocytic and neuronal factor whose expression correlates with glutamate toxicity but governs also neurogenesis. A transient elevation with significance at 7 dpl and also maximal levels at 14 dpl occurred for mainly SGZ-expressed *Npas3* (encoding Neuronal PAS Domain Protein 3, aka BHLHE12) as transcription factor that controls astrogenesis and neurogenesis. From 7-28 dpl, an upregulation with maxima at 14-28 dpl was observed for glial *Cdk5rap2* (encoding CDK5 Activator-Binding Protein C48, aka CEP215) as component of centrosomes and regulator of microtubule organization and neurogenesis. As a late induction from 14 dpl and maximal levels at 28 dpl, *Apoe* (encoding Apolipoprotein E), as reactive astrocyte-expressed membrane repair mediator and risk factor for Alzheimer’s disease together with Amyloid-Precursor-Protein, appeared to mirror the final resumption of OML activity.

**Remodeling response of the OML extracellular matrix**

ECM was rapidly modified in the OML after lesion, in line with published data (Deller et al., 2000). Entorhinal deafferentation was previously shown to trigger profound expression changes of ECM factors such as tenascin-C (Deller et al., 2000), which exhibited a chronic upregulation here with levels at maximum already at 1 dpl. A massive chronic upregulation with maximum at 3 dpl for *Glycam1* (encoding Glycosylation-Dependent Cell Adhesion Molecule 1) would modulate the initial adhesion of leukocytes to the regional endothelium that is followed by tissue infiltration. Enhanced ECM permeability for synapse repair and cell migration is presumably beneficial at this stage.

A strong response to the loss of Reelin secretion from entorhinal afferents was evident. Chronic massive downregulation to 14% with minimal levels at 3 dpl was documented for Adamts3 (encoding A Disintegrin and Metalloproteinase with Thrombospondin Motifs 3) as secreted metalloproteinase, which is responsible for Reelin degradation and therefore would promote sustained Reelin signaling, increase neurogenesis, and activate biogenesis and maturation of dendritic spines in newborn granule cells. In parallel, synapses would be adjusted also by the minor transient downregulation (at 3 dpl with minimal levels at 7 dpl) of the perisynaptic matrix component *Hapln4* (encoding Hyaluronan and Proteoglycan Link Protein 4). HAPLN4 is upregulated by excessive excitation in epileptic mouse brain, and serves in perineuronal nets together with neuronal SEMA3, matrix metalloproteases (MMPs), and with the transiently downregulated astrocytic *Vcan* (encoding Versican, localized particularly around parvalbumin-expressing neurons) to restrict signal diffusion.

**Remodeling responses of the OML axons and presynapses**

To establish whether factors have primary axonal roles, although cell cultures studies will always be biased towards detection in larger dendrites and neurites, we consulted a hippocampal RNAseq database where neuronal axons and somata were analysed separately from the dendrites and surrounding neuropil.

The neuronal tissue loss in the denervated OML decreases its original size by 30-50% (Phinney et al., 2004; Vuksic et al., 2011) and most neuronal transcripts in the OML showed similar decreases. It was interesting to note slightly progressive reductions of unspecific axon markers such as *Nefl* and the mainly interneuron expressed *Nefh* (encoding the Neurofilament light and heavy chains), decreased to 66-81% at 1-28 dpl, and 67-74% at 7-28 dpl, respectively, as structural correlates of impaired axonal length. Similarly, the chronic reduction of *Cntn4* to 63-70% (encoding Contactin-4, as glycosylphosphatidylinositol (GPI)-anchored or secreted promoter of neurite outgrowth, mediator of axonal target specificity and as Amyloid-Precursor-Protein interactor), reflected the regional axon loss. As contrasting example of clear temporal regulation, a delayed reduction with minimal levels at 7 dpl that disappeared by 28 dpl was observed for *Dock3* (encoding Dedicator of Cytokinesis 3, aka MOCA), as BDNF-dependent instructor of axon guidance, modulator of microtubule assembly and actin polymerization. This event reflects a process upstream from neurofilament organization and was best mirrored by the temporal downregulation of *Nefm*. Overall, minor chronic transcript reductions may not represent selective transcriptional downregulations but instead a mass phenomenon.

The period of lowest synaptic signaling appeared to cover 7-14 dpl, in view of the minimal levels of the immediate-early transcript *Nrn1* reductions to ~50% (encoding Neuritin-1, aka cpg15) as neuronal activity- and neurotrophin- dependent, GPI-anchored or secreted ligand that promotes axon branching. In temporal overlap, reductions to ~30% for *Ptpn3* (encoding the cytoskeleton-associated Protein-Tyrosine Phosphatase H1, crucial for (crucial for intracellular signal transduction from axon pathfinding receptor tyrosine kinases) mirror the peak neurotrophic depletion in the OML 1-2 weeks after injury. Somewhat later at 14-28 dpl, the expression reduction of *Fat3* to 47-65% (encoding the FAT atypical cadherin adhesion factor 3) as repressor of neuron migration, axon fasciculation and dendrite development presumably reflects the need to minimize neurite retraction and unipolarity at this stage. Given that *Fat3* mutation causes formation of excess neurites, its downregulation after ECL may contribute to denervation-induced axonal sprouting.

Presynaptic factors with downregulations reflected alleviated inhibitions. *Grm3* (encoding Metabotropic Glutamate Receptor 3, aka mGlu3, a member of group II metabotropic receptors) is present in presynapses but also in astrocytes, signals via G-proteins and inhibits adenylate cyclase. *Grm7* (encoding Glutamate Metabotropic Receptor 7, aka mGlu7, a member of group III metabotropic receptors) also signals via G-proteins and inhibits adenylate cyclase, to regulating axon outgrowth through the MAPK-cAMP-PKA signaling pathway. Mainly interneuron-expressed *Grm8* (encoding Metabotropic Glutamate Receptor 8, aka mGlu8, a member of group III metabotropic receptors) again signals via G-proteins and inhibits adenylate cyclase, to modulate anxiety-related signals. In addition, reduced transcript levels were documented for a presynaptic modulator of glutamate release and excitatory input to interneurons, which is known as *P2rx2* (encoding purinergic ATP receptor P2X2).

The entorhinal denervation triggered also downregulations of several modulators of vesicle priming to control neurotransmitter release in the active zone. This included firstly reduction to 48% for *Ppfia2* (encoding Liprin alpha 2, as clustering scaffold for the protein tyrosine phosphatases of receptor type and axon guidance molecule PTPRF, which targets ephrins, and ensures presynaptic efficacy in the active zone) with downregulation until 14 dpl. Further, this included a functional interactome, with reduced *Stxbp1* to ~60% (encoding Munc18-1, mainly interneuron-expressed) at 7-14 dpl, reduced *Unc13a* to 58-79% (encoding Munc13-1) in chronic manner, and reduced *Dgki* to 55% (encoding Diacylglycerol-Kinase Iota) at 14-28 dpl. Munc18-1 and Munc13-1 jointly control the quantal size of neurotransmitter vesicles in Diacylglycerol (DAG)-dependence, so presynapses may have altered release of neurotransmitter in response to the depleted number of excitatory afferents. Also, the chronic reduction of mainly interneuron-expressed *Snap91* (encoding 91 KDa Synaptosomal-Associated Protein, aka AP180 or CALM) provides evidence that the cycling of synaptic vesicles is affected. These reductions have an effect size that might be explained away by neuronal tissue loss. However, they occur only in specific time windows while most other presynaptic transcripts are unaffected, supporting the notion of active transcriptional downregulations. This deficit of multiple molecular switches in glutamatergic neurotransmission machinery is very interesting in view of electrophysiological data reporting a synaptic strengthening of surviving synapses in the dentate gyrus 3 days after ECL (Lenz et al., 2019). Reduction of presynaptic inhibition could contribute to synaptic upscaling (Wen and Turrigiano, 2024). Indeed, the metabotropic receptors of group II and III, *Grm3*, *Grm7* and *Grm8*, encode repressors of NMDA receptor activity (Ambrosini et al., 1995; Faden et al., 1997; Allen et al., 1999) and their deficiency would therefore explain the increased excitation of surviving entorhinal afferents.

Growth cone attraction efforts appeared to peak at 3-7 dpl. As one of the few factors that are upregulated in OML and GCL after entorhinal deafferentation, in delayed transient and putatively compensatory manner, mainly interneuron-expressed *Nrp1* (encoding Neuropilin-1, as coreceptor for the entorhinal axon chemorepulsant ligand Semaphorin 3E) appears to represent a lesion-specific upstream regulator, which is able to direct the reconnection of this specific circuitry via CDK signaling. The upregulation of growth-cone-enriched *Cyfip1* only at 3-7 dpl (encoding Cytoplasmic FMR1 Interacting Protein 1, aka SRA1) is relevant in this context. This factor was reported to serve as actin filament organizer and translation repressor of astrocytic focal adhesions and of neurons, regulating membrane ruffles and lamellopodia, promote dendritic complexity and spine maturation, influence axon formation, presynaptic size and neurotransmitter release probability. Thus, its multiple putative roles contrast with its induction in a very specific time window at the peak of neurite regeneration efforts, and with the redistribution of its mRNA preferentially to somata/axons according to the hippocampal RNAseq database. Overall, both upregulations of growth cone regulators might reflect cellular efforts to promote OML axon regeneration. Also, exclusively during this restricted phase, upregulation was documented for *Tmsb4x* (encoding Thymosin Beta-4) as actin-monomer sequestering factor that inhibits polymerization (reported to modulate cell migration, adherens junctions, and dendritogenesis, but redistributing preferentially to soma/axons in RNAseq).

**Remodeling response of OML neuronal somata**

The entorhinal deafferentation was reflected by downregulation of several factors, which have a somatodendritic distribution. As marker of granule cells, *Calb1* (encoding Calbindin-1) was reduced already on 1 dpl to 40% and remained significantly deficient until 7 dpl. This observation would mainly reflect granule cell dendrites in the OML, but denervation of CALB1-positive interneurons and semilunar granule cells (SGCs) might also contribute. As marker of neuronal activity loss, early transient downregulations until 7 dpl affected *Sphkap* (encoding SPHK1-Interactor and AKAP Domain-Containing Protein, aka SKIP), as converging factor linking cAMP and sphingosine signaling pathways, which couples excitation to Ca^2+^ activated PKA signaling, showing maximal deficit at 1 dpl that recovered steadily.

The transcriptome profile identified the putative nuclear coordinators of axonal re-orientation. Parallel early transient downregulations of transcription factors from 1 dpl until 14 dpl were documented for *Irx4* (encoding Iroquois Homeobox Protein 4), as transcription factor responsible for neural patterning and axon guidance cues expression, and for *Bhlhe22* (aka *Bhlhb5*, encoding Basic Helix-Loop-Helix Family, Member E22) as transcription factor responsible for axonal mistargeting. A converse acute and chronically sustained upregulation was observed for *Jun* (encoding c-Jun Proto-Oncogene and Activator Protein-1 Transcription Factor Subunit), known for its role in neural injury response and following entorhinal denervation (Haas et al., 1993; Haas et al., 1999)

In the period of minimal neuronal activity at 3 and 14 dpl, a transient downregulation concerned *Trim32* (encoding Tripartite Motif-Containing Protein 32), which was described as modulator of mTORC1-dependent growth, dendritic arborization, synaptic scaling, AMPA receptor endocytosis, but whose mRNA has a soma/axon localization in RNAseq data.

The denervation of interneurons was evident, in view of a chronic downregulation of *Gad1* to 54-73% (encoding Glutamate Decarboxylase 1, as the enzyme responsible for GABA neurotransmitter biosynthesis), an acute prolonged downregulation of *Gabra1* to 50% (encoding Gamma-Aminobutyric Acid (GABA) A Receptor Alpha 1), and a delayed reduction of *Pvalb* to 36-47% (encoding Parvalbumin, aka PV, in PV-positive interneurons). Overall, the reductions of *Calb1* and *Pvalb* indicate that early during the time course, the CALB1-positive granule cells and interneurons, and somewhat later also the PV-positive interneurons are strongly affected by dedifferentiation, in line with published reports (Nitsch and Frotscher, 1991; Nitsch et al., 1992; Nitsch, 1993).

**Remodeling response of the OML factors present both in dendrites and axons**

The fine-tuning of excitability was also modulated throughout neurites. A very notable transcript reduction to 30-62% was observed for *Vamp1* (encoding Synaptobrevin-1) until 28 dpl, and to 71-77% for *Prkcz* (encoding Protein Kinase C Zeta, aka PKMzeta) at 7 and 28 dpl, as VAMP kinase as well as potential axon sprouting modulator. Despite Synaptobrevin-1 and PKMzeta being associated in the literature mainly with axons and presynaptic vesicles, their mRNAs redistribute evenly between soma/axons and neuropil/dendrites/postsynapses in RNAseq, and neuronal Synaptobrevin has important roles also in the post-Golgi complex. Some late repair success was reflected in the levels of *Lrrc7*, which showed reduction to 81% initially at 1 dpl, and continued downregulated in the period of minimal synaptic activity at 7-14 dpl, but no longer at 28 dpl. LRRC7 was described as modulator of calcium channels, CAMK2, and glutamatergic receptors, as well as a regulator of dendrite branching. It is associated with cisternal stacks in the spines but also was observed in the axon initial segment.

Trafficking factors and membrane dynamics in neurites underwent specific modulations. Chronic reduction of *Kalrn* (encoding Kalirin) document deficient levels of axon guidance components. The RhoGEF KALRN protein is governed via Ephrin receptor activation and was described as regulator of AMPA and NMDA receptor homeostasis, as well as spine growth. However, its mRNA is redistributed more towards soma/axons rather than to dendrites according to RNAseq. Reductions to 61-70% at 3-14 dpl, and 55-76% at 3-28 dpl, respectively, were documented for *Kif5c* (encoding Kinesin Heavy Chain Neuron-Specific 2) and mainly interneuron-expressed *Kif5a* (encoding Kinesin Heavy Chain Neuron-Specific 1). Therefore, the transport capacity for neurotransmission machinery and for mRNAs may be decreased. The chronic reduction of *Agtpbp1* (encoding the Protein Deglutamylase CCP1, aka NNA1) as determinant of neurite microtubule transport also suggests that neuronal trafficking activity has not fully resumed by 28 dpl. The delayed reduction of mainly interneuron-expressed *Nrsn1* (encoding Neurensin-1), as vesicle-/microtubule-associated neurite extension factor at 3-28 dpl contrasted with a report on its induction after sciatic nerve crush injury (Suzuki et al., 2007), perhaps due to transiently impaired axonal flow. Indeed, the even further delayed downregulations of the retrograde microtubular motor *Dnalc1* (aka *Dnal1*, encoding the dynein axonemal light chain 1 protein) would reflect efforts to modulate anterograde trafficking at 7-28 dpl.

The upregulations concerned two small GTPases that promote microtubule-dependent intracellular vesicular transport. Firstly, *Arf4* (encoding ADP Ribosylation Factor 4, aka ARF2), as known axotomy-induced factor, dentate gyrus dendrite pattern regulator, and radial migration coordinator from 7-14 dpl. Secondly, *Rab8b* (encoding Ras-Related Protein Rab-8B) as dendritic membrane trafficking and exosome factor at 3-7 dpl and 28 dpl. Thus, membrane remodeling by vesicle regulators seems to be crucial as compensatory response after lesion.

**Remodeling response of OML dendrites**

The dysregulation of several dendrite modulators indicated that their repair is incomplete by the end of the observation period. This is in line with the literature, which indicates that remodeling of dendrites after entorhinal denervation occurs for several months postlesion (Vuksic et al., 2011).The chronic downregulation of *Btbd3* to 70% until 28 dpl (encoding BTB/POZ Domain-Containing Protein 3), as factor with abundant expression in granule cells including their dendrites upon ISH, whose transcription is neuronal activity-regulated (Wang et al., 2017), and which directs dendrites towards their innervating axons. BTB domains are known to modulate cytoskeletal mobility (Bomont et al., 2000). This observation may not only be explained by the OML tissue loss with axon depletion but may represent the secondary deficit in granule cell excitation.

An upregulation was found again for a small GTPase that promotes microtubule-dependent intracellular vesicular transport. Mainly interneuron-expressed *Arl4c* (encoding ADP-Ribosylation Factor-Like Protein LAK, aka ARL7), as modulator of hippocampal dendritogenesis showed increased levels across 1-28 dpl. Furthermore, upregulation from 7-28 dpl for *Cdk5rap2* (encoding Centrosomin, aka CDK5 Activator-Binding Protein C48) as microtubule extension factor acts within the same pathway given that CDK5 is responsible for the phospho-regulation of small GTPases. Jointly, these data suggest that dendrites compensate for deficient stimulation by remodeling their branches via enhanced microtubular vesicle transport.

**Remodeling response of OML excitatory and inhibitory postsynapses and spines**

The data identified *Neto1* as selectively and strongly affected spine factor. The massive downregulation to 39-59% for *Neto1* (encoding Neuropilin and Tolloid Like 1, aka BTCL1), presumably affected it as transmembrane factor containing a peptidase domain and a LDL receptor domain, because it acts as potential SEMA3E receptor, according to the STRING interaction database. This observation is consistent with the loss of 30-40% of dendritic spines following ECL (Vuksic et al., 2011). It is mainly expressed in somatostatin-, cholecystokinin/cannabinoid receptor 1-, and parvalbumin-containing interneurons, known as modulator of NMDA and kainate receptors, and as DLG2/3/4-interactor.

More moderate downregulations that might be explained by OML tissue loss were affecting the following factors. *Dlg2* (encoding Discs Large MAGUK Scaffold Protein 2, aka Chapsyn-110, aka PSD93), as modulator of NMDA receptors that links excitotoxicity with neuroinflammation; *Cnih2* (encoding Cornichon homolog 2), as modulator of AMPA receptor membrane assembly and desensitization; *Homer2* (encoding Homer protein homolog 2, aka Cupidin, aka VESL-2), as postsynaptic density scaffold, effector of metabotropic glutamate receptors, and drebrin interactor; *Gria1* (encoding the Glutamate Ionotropic Receptor AMPA Type Subunit 1, aka GluA1 or GluR1 or GluR-A), as main mediator of fast excitation and of long-term potentiation during synaptic plasticity. All these downregulations represent deficient glutamatergic neurotransmission components.

Several factors in general postsynapses independent from glutamatergic excitation were affected. Moderate downregulations were detected for the following factors. *Ddn* (encoding Dendrin), implicated in cytoskeleton, CIN85-mediated endocytosis, and retrograde signaling from synapse to the nucleus; *Rxfp1* (encoding the Relaxin Family Peptide Receptor 1, aka LGR7), which acts via G proteins and activates a tyrosine kinase pathway to stimulate adenylate cyclase and increase cAMP, as well as to generate nitric oxide; *Lypd6* (encoding Ly6/PLAUR Domain-Containing Protein 6), as mainly interneuron expressed, GPI-tethered modulator of nicotinic acetylcholine receptors, and positive regulator of Wnt/beta-catenin signals.

Upregulations of postsynaptic factors were observed in chronic manner for *Gabarap* (encoding GABA Type A Receptor-Associated Protein, aka ATG8A) in chronic manner as a factor which is primarily known to cluster GABA neurotransmitter receptors while linking them to the cytoskeleton, but was also shown to modulate autophagy, and play a role at the axon initial segment. This may reflect a longstanding signal repression during tissue remodeling. In contrast, the upregulation of *Lyn* (encoding the Tyrosine-Protein Kinase and Proto-Oncogene Lyn) occurred in delayed manner and disappeared by 28 dpl, suggesting that its role as mediator of neuronal polarization and as amplifier of glutamate signals is completed.

**II. Transcriptional adjustments occurring in the GCL**

At the systems biology level, GSEA software in the GCL showed the following: The entorhinal deafferentation firstly triggered downregulations (Fig. 4) at 1 dpl for axonal conductance (GOCC term axon initial segment), and in sustained manner for presynaptic and dendritic excitation (GOCC terms presynaptic active zone, synaptic vesicle, dendritic shaft, mossy fiber to CA3 synapses, neuron projection) and neuronal respiration (GOCC terms mitochondrial complex, respirasome, cytochrome complex), in sustained manner between 3-14 dpl. The impaired neural activity appeared to include also the translation machinery at the 7 dpl peak (GOCC term organellar ribosome). The reduction of signaling factors was evident at 1-7 dpl (GOMF terms ion, receptor binding, channel activity, pheromone binding). Again, at the 7 dpl peak, the impairment of synaptic transmission and respiratory complex-I activity became detectable (GOBP terms synaptic vesicle priming and NADH-dehydrogenase complex assembly).

The entorhinal deafferentation secondly triggered upregulations (Fig. 4) of protein synthesis factors (GOCC terms for cytosolic ribosomes, polysomes) from 3 dpl onwards. This was accompanied by elevated demand for translation control factors and for rRNA binding molecules (GOMF terms) at the 7 dpl peak. As presumptive repair effort, synaptic translation was found increased at 3-7 dpl (GOBP terms). These general pathway effects are as expected in brain tissue under reorganization, with additional information on their temporal coordination included in the datasets.

In analogy to OML findings of low semaphorin (Sema3e/Sema5a) and altered neuropilin (Nrp1 up / Neto1 down), prominent among GCL downregulations were the transient reduction of Nrp2 versus induction of Nrp1, as well as decreased Epha5, Epha7 and Epha6, together with Usp33, as axon growth and cell migration factors. Also conspicuous, regarding general neurotrophic signaling, was the diminished expression of Bdnf and Hgf that encode ligands, accompanied by lowered Egfr, Erbb4 and Igf2r mRNAs that encode the corresponding tyrosine kinases and receptors, providing a credible explanation for the dedifferentiation of local neurons.

As previously done in the section on the OML, a detailed consideration of individual findings was attempted in separate paragraphs with subtitles below, grouping the transcript dysregulations according to the neuronal compartments, temporal co-regulations and functional pathways which they help to adjust. Again, the relevant literature for each factor is supplied in the Supplemental Table as PubMed-IDs.

**Remodeling response of glia in the GCL**

In contrast to OML tissue where the lesion had caused axon degeneration and tissue loss, in the GCL no activation of Gfap/Vim in astrocytes or Aif1/Tyrobp in microglia was found. Instead, several glial factors that coordinate stem cells showed temporal expression changes, supporting the concept that entorhinal denervation alters neurogenesis in the dentate gyrus (Cameron et al., 1995), although this has been controversial (Fontana et al., 2006).

Immediate chronic upregulation with maximal levels at 1 dpl and 28 dpl was observed for *Rnf213* (encoding the E3-ubiquitin-protein-ligase Mysterin). *Rnf213* acts as proteolysis mediator and it was previously associated with neuroinflammatory reactions to brain damage. Its upregulation may be linked to (micro)glial activation according to RNAseq data, although one report claimed its transcriptional induction exclusively in neurons after ischemia. A strong and chronic upregulation with maximum at 14 dpl stood out for *Nup210l* (encoding Nuclear Pore Membrane Glycoprotein 210kDa-Like, predicted as single-pass transmembrane protein), which is expressed preferentially in glial and vascular cells as well as in neural progenitors, where it shows converse regulation to the stem cell differentiation factor *Nup133*, again suggesting that neural stem cell (NSC) differentiation can be modulated and restricted by glia at this late stage. In similar manner, chronic elevation of *Lmna* (encoding Lamin-A or Lamin-C, in dependence on alternative splicing, as major nuclear envelope constituents and intermediate filaments) was noted. These factors are present in any cell but downregulated in postnatal brain. Lamin-A expression in brain was previously recognized as marker of (astro)glial proliferation or vascular infiltration or ageing stress. The induction of *Notch2* (encoding Neurogenic Locus Notch Homolog Protein 2, a membrane receptor) was significant in the late period 7-28 dpl while regeneration and differentiation are ongoing. NOTCH2 is selectively expressed in NSC and its signaling is known to maintain NSC quiescence while supporting radial glia.

At the level of nuclear expression coordination, the converse immediate and chronic downregulation with minimal levels already at 1 dpl for transcription factor *Nfia* (encoding Nuclear Factor IA, aka CCAAT-Box-Binding Transcription Factor, or TGGCA-Binding Protein), whose deficiency results in the conversion of glia into neurons, the scenario is compatible with cooperative control over neural reprogramming. The chronic induction with maximum at 3 dpl for *Setd1a* (encoding the Histone Lysine Methyltransferase SET Domain Containing 1A), as nuclear factor that ensures NSC quiescence, supported the concept of altered regulation of stem cells in the dentate gyrus. Downstream, the chronic induction with maximum at 3 dpl for immediate-early factor *Nov* (encoding Nephroblastoma Overexpressed Gene, aka Cellular Communication Network Factor 3 or *Ccn3*, or *Igfbp9*) as putative interactor of NOTCH and as secreted regulator of matrix metalloproteases may represent an effector in this pathway.

Later, upregulation at 14-28 dpl was documented for *Sip1* (encoding Smad Interacting Protein 1, aka Zinc Finger E-Box Binding Homeobox 2 or ZEB2 or ZFHX1B) as master regulator of Bergmann astrocyte development / astrogliosis / Schwann cell differentiation, with a repressor role for the expression of EphrinB2, Semaphorin3F, E-cadherin and other genes implicated in neurogenesis and migration. In sum, there is no evidence of increased glial mass in the GCL, but the datasets indicate a change of differentiation in microglia, astrocytes and effects on dentate gyrus stem cells.

**Remodeling response of the GCL extracellular matrix (ECM)**

In perfect alignment of OML and GCL findings, the levels of the Reelin inactivation factor *Adamts3* were again downregulated. In the GCL, this occurred only at the beginning and end of the observation period, with smaller effect sizes. This may contribute to the reprogramming of stem cells to astrocytes or to newborn granule neurons immediately after lesion in a cooperation of Reelin and Notch signaling (Hashimoto-Torii et al., 2008), while later on supporting their migration, neurite extension, and excitability. A transient downregulation at 3-7 dpl was found for *Prss12* (encoding Serine Protease 12, aka Neurotrypsin or Motopsin), as secreted brain-specific neuron-expressed member of its gene family, and with soma/axon distribution according to RNAseq data, which promotes neurogenesis and immature spine numbers. *Mmp3* (encoding Matrix Metallo-Proteinase 3), a member of the large family of metalloproteinases that play a role in numerous cell signaling processes through extracellular matrix molecules, growth factors, and receptors, demonstrated robust downregulation at 3-28 dpl. Overall, the transcriptional downregulation of three proteases (*Adamts3*, *Prss12, Mmp3*) and two NSC quiescence factors (*Notch2*, *Setd1a*) points to a remodeling of the ECM in the GCL, although this region was not directly affected by the surgery.

**Remodeling response of excitatory and inhibitory postsynapses and spines**

Temporally specific deficits of spine adhesion factors were evident in the GCL. Among the downregulated genes, two glycosylated synaptic cell adhesion molecules, i.e. mainly postsynaptic *Lrrtm3* (neutral distribution between neuropil and soma, encoding Leucine Rich Repeat Transmembrane Neuronal 3) and possibly presynaptic *Lrrtm1* (distributed to soma/axon fraction upon RNAseq)**,** which are known for their essential role in excitatory synapse development and function, were altered only at 1-3 dpl for *Lrrtm3*, but during 1-28 dpl for *Lrrtm1*. It is noteworthy that overexpression of *Lrrtm3* was demonstrated to enhance excitatory synapse density in dentate granule neurons, whereas its knockdown reduces it. Moreover, *Lrrtm3* regulates the activity-dependent expression of AMPA receptors. Acute transient downregulation at 1-3 dpl was documented also for *Nrp2* (encoding Neuropilin 2, aka Receptor for VEGF165 and Semaphorins Class 3) as SEMA3E binding factor, modulating dendrite branching and spine number / size as basis of memory and motor functions. Downregulation from 7-28 dpl was documented for *Pcdhb16* (encoding Protocadherin Beta 16, aka PCDH3X) as calcium-dependent cell adhesion protein, involved in establishment and maintenance of specific neuronal connections.

The expression of several interneuron factors was weakened transiently. Downregulation at 3-7 dpl was documented for DLG4/PSD95-associated but mainly interneuron-expressed *Erbb4* (encoding Erb-B2 Receptor Tyrosine Kinase 4, aka HER4 or ALS19), as trophic hub where NRG3 / NRG4, other neuregulins, EGF, epiregulin and betacellulin can bind as ligands, to modulate neurogenesis, neurite outgrowth, dendritic arborization, neurotransmission in GABAergic cells and their excitatory input to influence long-term potentiation and gamma-oscillations in the hippocampus. As additional regulator of GABAergic interneurons and social behavior, *Avpr1a* (encoding Arginine Vasopressin Receptor 1A, aka Vasopressin V1a receptor) also exhibited immediate transient downregulation at 1-3 dpl.

Converse upregulation from 3 dpl was observed for *Ephb1* (encoding the Ephrin B1 Receptor), which is expressed in hippocampal neural progenitors with GABAergic fate, acting in Reelin-SRC-dependent manner on MAPK-Jun signaling to modulate neurogenesis, polarity, growth cone collapse, glutamatergic excitation, and activation of astrocytes or oligodendrocytes. *Ephb1* induction was reported before, in an entorhinal deafferentation study where immunohistochemistry indicated that this response originated in reactive astrocytes in the first week after lesion (Wang et al., 2005b). Upregulation in chronic stable manner was documented for *Dlg4* (encoding Discs Large MAGUK Scaffold Protein 4, aka SAP90 or PSD95), as postsynaptic density component, and interactor of CNKSR2, presumably as a cellular effort towards increased synaptic strength.

Cellular efforts to compensate the neurotrophic state were conspicuous and constitute potential targets for therapeutic interventions. The chronic inductions with maximum at 7 dpl for *Sorcs2* (postsynapse/neuropil distribution in RNAseq) and maxima for 1 dpl as well as 14 dpl for *Sorcs3* (soma distribution in RNAseq) are noteworthy. *Sorcs2* (encoding Sortilin-Related VPS10 Domain Containing Receptor 2) was demonstrated to function as coreceptor of the neurotrophic receptors p75(NTR) (aka NGFR) and TRKB (aka NTRK2) in hippocampal neurons, and to transduce also e.g. progranulin signals for motor neuron axonal outgrowth. ProNGF or ProBDNF ligand binding triggers growth cone collapse, inactivation of RAC1/RAC2, and reorganization of the actin cytoskeleton. SORCS2 controls dendritic spine density, synaptic plasticity and fear extinction. Parallel chronic increase was found for *Sorcs3* as trophic coreceptor, which is mainly interneuron-expressed, and shows localization to vesicles in soma and dendrites. Dentate *Sorcs3* expression is induced within hours after seizures, and SORCS3 protein interacts with DLG4 (aka PSD-95 or SAP-90), is responsible for glutamatergic function in spines with postsynaptic depression and attenuates BDNF signaling to modulate energy balance as well as orexigenic peptide production. This gene family is encoded by many small exons with exceptional regulatory possibilities, so they belong to the largest known genes, and the entire SORCS protein family was strongly associated with Alzheimer’s disease upon genome-wide studies.

Downstream from neurotrophic signaling, delayed transient upregulation at 3-14 dpl was observed for the c‑Abl- or injury-inducible immediate-early transcript *Gem* (encoding GTP Binding Protein Overexpressed In Skeletal Muscle, aka Kinase-Inducible Ras-Like Protein KIR) as calcium-responsive, GTP-binding, negative regulator of Rho kinase-mediated cytoskeletal reorganization (stress fiber formation and neurite retraction) and inhibitor of voltage-gated calcium channel activity, to modulate receptor-mediated signal transduction to the nucleus, neurite growth, dendritic morphology and cell shape. Chronic upregulation with a maximum at 28 dpl occurred for *Ablim3* (encoding Actin Binding LIM Protein Family Member 3) as cytosolic actin-binding DYRK1A-phosphorylated scaffold protein with the ability to modulate transcriptome programs, which serves in the Netrin control of neurite outgrowth (possibly as downstream modulation of decreased *Ntng1* levels in the GCL, see above), and which ensures granule cell connectivity.

**Remodeling of GCL dendrites**

Several transcripts with distribution to the neuropil upon RNAseq and functions in dendrites showed significant changes, most of which had decreased expression. Chronic downregulation with minimum at 3 dpl occurred for seizure-repressed *Nrep* (encoding Neuronal Regeneration Related Protein, aka P311 or PTZ-17) as Rho kinase antagonist, activator of migration and neurite outgrowth/regeneration via SMAD signaling. Downregulation at 3-7 dpl occurred for NOTCH-inducible *Fjx1* (encoding Four Jointed Box 1) as the rodent ortholog of the *Drosophila* planar cell polarity (PCP) protein Four-jointed (Fj), which was found to be a Golgi kinase, promoter of FAT3 signals, and inhibitor of dendrite extension. Interestingly, hippocampal neurons from *Fjx1* mutant mice showed an increase in dendrite extension and branching, whereas the addition of *Fjx1* had the opposite effect, reducing dendrite length and decreasing dendritic branching**.** Chronic downregulation with minimum at 28 dpl was found for *Mtap2* (encoding Microtubule Associated Protein 2, aka MAP2) as factor that stiffens microtubules and stabilizes dendritic shape. These factors appear to modulate dendrite trafficking. Conversely, upregulation at 1-3 dpl was found for interneuron-expressed *Rab17* (encoding Ras-Related Protein Rab-17) as small GTPase and mediator of transcytosis, dendrite morphogenesis and postsynaptic development.

**Remodeling response of GCL neurites**

Several transcripts with balanced distribution between neuropil and soma upon RNAseq, and with functions both in dendrites and axons also showed significant changes. Chronic downregulation with minimum at 1 dpl occurred for *Grik2* (encoding Glutamate Ionotropic Receptor Kainate Type Subunit 2, aka GluR6) as excitatory signal transduction amplifier, expressed in dentate granule cells, mediating slow-deactivating currents, as modulator of synaptic plasticity and seizure vulnerability. Chronically decreased levels with minimum at 7 dpl were found for *Ntng1* (encoding Netrin-G1) as marker of mature granule cells, a secreted axon and dendrite guidance factor, modulating excitatory synaptogenesis. Downregulation at 1-14 dpl was observed for the *Glra2* (encoding the Glycine Receptor Alpha‑2) as inhibitory signal transduction molecule, expressed in adult neural stem cells as essential factor for cortical progenitor homeostasis, interneuron migration and neuronal projections. Downregulation from 1-14 dpl was observed in the GCL, as before in the OML, for *Lrrc7* (encoding Leucine Rich Repeat Containing 7, aka Densin-180 or LAP1). According to the STRING database LRRC7 interacts with the postsynaptic scaffold CNKSR2, with both factors showing similarly chronic downregulation. LRRC7 as binding partner of Cadherin / Catenin is implicated in AMPA/NMDA-modulation, spine biogenesis, and dendrite branching. Interestingly, LRRC7 is also found in the axon initial segment. Downregulation at 7-14 dpl was documented for *Cabp1* (encoding Calcium Binding Protein 1, aka Caldendrin), which directly couples postsynaptic calcium signaling to actin remodeling in dendritic spines, via interaction with myosin V. Interestingly, caldendrin plays a role in the localization of the spine apparatus organelle (Konietzny et al., 2023) a specialized form of endoplasmic reticulum (ER) that is crucial for Hebbian and homeostatic synaptic plasticity in granule cells (Deller et al., 2003; Vlachos et al., 2013). Following entorhinal denervation, the spine apparatus is resorted within the granule cell dendritic tree (Deller et al., 2006). In sum, these changes appear to be consistent with a reduced excitatory and inhibitory neurotransmission during the first half of the observation period.

Compensatory changes were also identified: Chronic upregulation with maximum at 1 dpl concerned *Gpr68* (encoding G Protein-Coupled Receptor 68) as detector of membrane-stress and shear-stress via its proton-sensing capacity, whose activity mirrors actin polymerization, being required for long-term potentiation and passive avoidance behavior, and acting via CREB and BDNF. Chronic upregulation with maximum at 14 dpl involved interneuron/astroglia-expressed *Cacng5* (encoding a Calcium Voltage-Gated Channel Auxiliary Subunit Gamma 5, aka Transmembrane AMPAR Regulatory Protein Gamma-5, a paralog of Stargazin) as PSD95/DLG4 interactor and AMPA modulator. Upregulation from 7 dpl with maximum at 28 dpl was found for *Marcksl1* (encoding Myristoylated Alanine-Rich C-Kinase Substrate Like 1, or MacMARCKS), which is induced after brain injury as determinant of actin stability and mediator of spine formation. Thus, the upregulated factors in GCL neurites in the regeneration / differentiation stage are modulators of AMPA receptors and actin fibers.

**Remodeling response of GCL somata**

As upstream controls over neuronal responses to entorhinal denervation, factors of retrograde feedback to the nucleus and transcription factors were prominent, exhibiting consistent changes following lesion. The chronically decreased levels with minimum at 1 dpl for *Calb1* (encoding Calbindin-1) as granule cell marker suggest that their differentiation state is impaired. Acute downregulation only at 1-3 dpl was noted for ubiquitous *Atxn10* (encoding Ataxin-10, aka SCA10) as interactor of the G Protein Subunit Beta 2 (GNB2), activator of Ras-MAPK signaling, regulator of cytokinesis, mediator of neuritogenesis, neuron differentiation, and neuron survival. Chronic downregulation with minimum at 7 dpl for *Mapk1* (encoding the Mitogen-Activated Protein Kinase 1, aka ERK2 or Extracellular Signal-Regulated Kinase 2) represented the lowest neuronal activity. Chronic downregulation with minimum at 14-28 dpl was also documented for *Cnksr2* (encoding Connector Enhancer of Kinase Suppressor of Ras 2, aka CNK2, or KSR2, or MAGUIN for Membrane-Associated Guanylate Kinase-Interacting Protein), which serves as scaffold for AMPA receptor assembly in postsynaptic complexes together with neurotrophic receptors / LRRC7 / DLG4. *Cnksr2* also mediates the coupling of Ras-dependent signal transduction to membrane/cytoskeletal remodeling, thus enabling the scaling of excitability, spine biogenesis, Rac cycling, Mapk/Notch signaling, as well as granule cell maturation. Downregulation from 3 dpl with minimum at 7 dpl concerned interneuron-expressed *Vsnl1* (encoding Visinin Like 1, aka VILIP or Hippocalcin-like Protein 3) as modulator of adenylyl cyclase signaling after calcium-dependent membrane association, which governs neuronal excitability scaling.

The most significant and extraordinarily massive downregulation (with minimum <1% at 28 dpl) regarded retrograde signaling factor *Rgs13* (encoding Regulator of G-Protein Signaling 13), known to be abundant in the dentate gyrus and hippocampal CA regions. The PKA-dependent RGS13 protein is a GTPase-activator for Galpha(i) and Galpha(o) to repress MAPK activity, acting in the nucleus to inhibit CREB-mediated gene expression.

Downregulations of several trophic factors correlated with the phase of minimal neuronal activity at 7-14 dpl. Such transient downregulation was documented for *Igf2r* (encoding Insulin Like Growth Factor 2 Receptor) as regulator of cell growth and spine maturation, also for *Egfr* (encoding the Epidermal growth factor receptor, aka ERBB1), which is mainly expressed by interneurons according to Allen Mouse Atlas ISH entry 81599632, and whose ligands EGF and Neuregulins promote neural stem cell activation and neurogenesis, and also for transcription factor *Hlf* (encoding Hepatic Leukemia Factor), known to restrain miniature excitatory responses upon prolonged activity deprivation, as member of the PARbZIP family controlled by the circadian molecular oscillator.

In contrast, as postulated mediator between the lesioned afferents and the trophic state of stimulated neurons, the levels of *Bdnf* (encoding Brain Derived Neurotrophic Factor), expressed in interneurons more than granule cells, as a master regulator of differentiation and dendritic architecture, were chronically diminished at 1-28 dpl with minimum at 28 dpl. It should be acknowledged that an *in-situ* hybridization study previously reported transient upregulation at 4 h after ECL (Forster et al., 1997). In this study, however, electrolytic entorhinal lesions were performed (mechanical lesions were performed in our study) and it could be shown that the increase in BDNF was activity-dependent (Forster et al., 1997).

Regarding the nuclear coordinators of transcription, a chronic downregulation with minimum at 1 dpl was observed for *Smarca2* (encoding SWI/SNF Related BAF Chromatin Remodeling Complex Subunit ATPase 2, aka Brahma or BRM) as nuclear helicase and component of the neural progenitors-specific chromatin remodeling complex (npBAF complex) and the neuron-specific chromatin remodeling complex (nBAF complex). Finally, chronic downregulation with minimum at 3 dpl was documented for *Gtf2h5* (encoding General Transcription Factor IIH Subunit 5, aka TFIIH subunit 8 or TTDA) as component of the transcription initiation complex whose levels modulate neural dysfunction in *C. elegans*.

Several membrane dynamics factors exhibited decreased levels. Downregulation at 3-7 dpl was observed for interneuron-expressed *Snap47* (encoding Synaptosome Associated Protein 47) as interactor of Syntaxin-17, component of the default autophago-lysosomal pathway, which controls BDNF release, and acts as excitatory synapse assembly factor. Downregulation at 3-7 dpl was also found for *Stxbp5* (encoding Syntaxin-1 Binding Protein 5, aka Tomosyn), as regulator of RhoA GTPase activity, which has been shown to play a critical role in stabilizing dendritic arborization and to control surface expression of AMPA receptors. Chronic downregulation with minimum at 3 dpl was found for *Arhgap12* (Rho GTPase-activating protein 12) as synaptic RhoGAP that regulates excitatory synaptic structure and function during development. Chronic downregulation with minimum at 7 dpl occurred for interneuron-expressed *Lin7b* (encoding Lin-7 Homolog B Crumbs Cell Polarity Complex Component, aka VELI2 or MALS2), which maintains the asymmetric distribution of channels and receptors at the plasma membrane of polarized cells, and interacts with Rho effectors to ensure polarization. LIN7B protein can be localized in a presynaptic complex with Munc18 or in a postsynaptic complex with PSD95/DLG4, to regulate NMDA receptors and Erbb4 receptor tyrosine kinase. Chronically decreased levels with minimum at 14 dpl were found for *Ralgps2* (encoding a guanine nucleotide exchange factor for the small GTPase RALA), which cooperates with the exocyst complex to regulate integrin-dependent exocytosis of membrane rafts, of dense core vesicles for protein secretion, and growth signaling. The RALGPS2 protein interacts with neurotrophic molecules and MAPK signals to modulate neural differentiation and induce *c-Fos* expression. Chronic downregulation with minimum at 28 dpl was observed for *Vps41* (encoding Vacuolar Protein Sorting-Associated Protein 41 Homolog, aka SCAR29), a factor required for TFEB-dependent regulation of the lysosomal-autophagic pathway via interaction with Syntaxin-17.

Several membrane adhesion factor transcripts with distribution to the soma upon RNAseq exhibited decreased levels. Chronic downregulation with minimum at 7 dpl concerned *Adam11* (encoding A Disintegrin and Metalloproteinase Domain Cysteine-Rich Protein 11) as integrin-binding, glycosylated integral transmembrane protein implicated in axon guidance. Chronic downregulation with minimum at 28 dpl was documented for *Adam23* (encoding A Disintegrin And Metalloproteinase Domain Cysteine-Rich Protein 23, aka MDC3) as integrin-binding, glycosylated integral transmembrane protein implicated in neuronal differentiation, axon guidance, the axon initial segment, excitatory neurotransmission and epilepsy. Chronic downregulation with minimum at 28 dpl occurred also for interneuron-expressed *Cadm2* (encoding Cell Adhesion Molecule 2, aka SynCAM2) as glycosylated integral transmembrane protein from the immunoglobulin superfamily, interactor of Nectin-like family, which is involved in energy homeostasis, axon pathfinding, axon-axon contacts, executive function and processing speed.

Several membrane receptor transcripts with distribution to the soma upon RNAseq exhibited decreased levels. Chronic downregulation with minimum at 3 dpl was observed for interneuron-expressed *Gabra1* (encoding Gamma-Aminobutyric Acid Type A Receptor Subunit Alpha1) as NOTCH- and seizure-dependent mediator of inhibitory neurotransmission. Chronic downregulation with minimum at 3 dpl concerned also interneuron-expressed *Gabra2* (encoding Gamma-Aminobutyric Acid Type A Receptor Subunit Alpha2) as anxiety-related mediator of inhibitory neurotransmission, which signals cell-autonomously during development to control positioning of newborn neurons, regulates late maturation of their dendritic tree, and ensures synaptogenesis, while remaining expressed in adult granule cells also to modulate repression by GABAergic interneurons upon altered excitation. Downregulation from 14 dpl to minimum at 28 dpl occurred for interneuron-expressed *Gabrb2* (encoding Gamma-Aminobutyric Acid Type A Receptor Subunit Beta2) as necessary but not sufficient factor to induce rapid synaptic contacts, and as extrasynaptic mediator of tonic GABAergic inhibition.

Converse upregulation was documented for several nuclear coordinators of expression. Upregulation at 1-3 dpl occurred for *Pitx3* (encoding Paired Like Homeodomain 3) as transcription factor regulating BDNF expression. In mice and *C. elegans*, a *Pitx3* ortholog cooperates with an *Adamts3* ortholog to determine GABAergic versus dopaminergic neuron differentiation. Upregulation at 7-28 dpl concerned *Tspyl2* (encoding Testis-Specific Protein Y Encoded-Like 2, aka CASK-Interacting Nucleosome Assembly Protein or CINAP, and Differentially-Expressed Nucleolar TGF-Beta1 Target Protein or DENTT, and Cell Division Autoantigen 1 or CDA1), as component of the CASK/TBR1/TSPYL2 transcriptional complex in interaction with CREB-binding protein, which modulates gene expression e.g. of neurotrophic factor *Bdnf,* NMDA glutamate receptors *Grin2a/b/c*, and *Prss16* as paralog of Prss12, in response to neuronal synaptic activity.

Regarding the amplification of neuronal signals, chronic upregulation with maximum at 1 dpl was found for neuron-specific Stac (encoding Src Homology Three (SH3) And Cysteine Rich Domain), which promotes membrane integration of voltage-gated calcium / sodium channels and slows their inactivation. Chronic upregulation with maximum at 28 dpl occurred for *Akap1* (encoding A-Kinase Anchoring Protein 1, aka AKAP149 or AKAP121 or S-AKAP84), which binds to regulatory subunits of PKA and anchors them to ER membranes surrounding the nucleus or mitochondria, to control the expression of LMNA (Lamin A/C) and to coordinate local protein synthesis at the mitochondrial surface, serving roles of cell growth, neuronal morphogenesis and neuroprotection against excitotoxicity. Upregulation from 14 dpl and maximum at 28 dpl was observed for *Stim2* (encoding Stromal Interaction Molecule 2), a transcript distributed to soma/axon upon RNAseq, which acts as NOTCH-dependent endoplasmic reticulum Ca2+ sensor to govern neuronal calcium homeostasis e.g. in presynapses and also modulates glutamate receptor activity.

Concerning Reelin signals from lost entorhinal afferents, upregulation from 3 dpl with maximum at 28 dpl occurred for interneuron-expressed *Nrp1* (encoding Neuropilin-1) as transcript with soma distribution. NRP1 protein specifically binds to the C-terminal region of Reelin and acts as a coreceptor for VLDLR (very-low-density lipoprotein receptor). Interaction of the NRP1/VLDLR protein complex with Reelin was shown to be essential for normal dendritic development in superficial layer neurons of the neocortex. Thus, we identified at least two dysregulated molecules, i.e. downregulated *Adamts3* and upregulated *Nrp1*, which act via the canonical Reelin pathway to adjust the GCL to denervation.

Upregulation at 7-14 dpl occurred for the Flrt3 transcript redistributed to the soma according to RNAseq (encoding Fibronectin Leucine-rich repeat transmembrane protein) as glycosylated integral membrane protein with shedded ectodomain, which serves neuronal migration / adhesion / repulsion, mediates Netrin‑1 responsiveness, acts as latrophilin ligand during spine development, and shows preferential expression in granule cells of the dentate gyrus. Chronic upregulation from 7 dpl and maximum at 14 dpl was observed also for *Itsn2* (encoding Intersectin-2) as link between endocytic membrane traffic and actin assembly machinery, modulating vesicle transport and activating signaling pathways, in connection with EphB2 and dependence from EGFR. Chronic upregulation with maximum at 14 dpl was also found for interneuron-expressed *Chrnb2* (encoding the Neuronal Nicotinic Acetylcholine Receptor Beta‑2), a transcript distributed to the soma fraction in RNAseq, which can influence neuronal progenitor differentiation.

**Remodeling response of the GCL axon initial segments**

Following entorhinal denervation of the dentate gyrus, significant expression changes for AIS-associated factors that control efferent signaling were found in the GCL, with a majority of downregulations. Notably, several genes associated with the structural integrity of the AIS, e.g. *Nrcam* (encoding Neuronal Cell Adhesion Molecule, aka Bravo) and *Ank2* (encoding Ankyrin 2, aka ANKB), showed a decrease in their expression levels (with minimum at 1 dpl and 3 dpl, respectively) suggesting early remodeling of the AIS. In addition, downregulations occurred for factors that are critical for transport and clustering of specific channels to the AIS, including firstly the mainly interneuron-expressed *Kif5a* (Barry et al., 2014) with minimal levels in OML at 7 dpl, secondly the mainly interneuron-expressed *Scn1a* (encoding Sodium Voltage-Gated Channel Alpha Subunit 1), with minimal levels in GCL at 7 dpl, and thirdly *Dlg2* as membrane-associated guanylate kinase and scaffold e.g. for potassium channels, with minimal levels in OML at 14 dpl, and in GCL at 28 dpl. Furthermore, several transcripts for ion channels and receptors were also downregulated, e.g. *Gabra2* with minimal levels in GCL at 3 dpl, which plays an important role in regulating GABAergic control of neuronal excitability in the AIS. Interestingly, the downregulation of GABA(A) receptors has also been observed in the AIS of pyramidal cells in specific areas of the prefrontal cortex in autism (Hong et al., 2020).

Converse upregulation from 3 dpl with maximum at 14 dpl was observed for *Mtcl1* (encoding Microtubule Crosslinking Factor 1, aka SOGA2), which was demonstrated to play an important role for the maintenance of the AIS in Purkinje cells. Thus, this gene may have a pivotal role for the stabilization of the AIS and for supporting recovery and a return to normal firing rates following lesion (Reeves and Steward, 1988).

**Remodeling response of the GCL axons and presynapses**

As earliest injury response with relevance for GCL axons, Usp33 (encoding Ubiquitin Specific Peptidase 33, aka VDU1) showed downregulation until 7 dpl with a minimum at 3 dpl. This factor acts upon nutrient starvation to trigger general autophagy instead of targeted phagocytosis, has also a regulator role for Golgi transport, and for ROBO1/SLIT signaling. Chronic downregulation was observed for several axon guidance family members, but in successive stages: Importantly, interneuron-expressed *Epha6* with a minimum at 1 dpl, *Epha7* with a minimum at 7 dpl, and *Epha5* with a minimum at 28 dpl (encoding Ephrin Tyrosine Kinase Receptors type A 6/7/5) as axon guidance factors and TRKB interactors showed consistent decreases.

Chronic downregulations with a minimum at 7 dpl in good correlation with the peak of repair concerned the following factors: *Grm7* (encoding Glutamate Metabotropic Receptor 7, aka MGLUR7) as presynaptic inhibitory modulator of trophic signaling and regulator of axonal outgrowth and nerve terminal development. *Basp1* (encoding Brain Abundant Membrane Attached Signal Protein 1, aka NAP22 or CAP23) as presynaptic factor with channel activity that regulates axon guidance, neuroregeneration and synaptic plasticity. *Syt17* (encoding Synaptotagmin 17, aka B/K Protein), with transcript distribution upon RNAseq to soma/axon and with inducibility after seizure, as PKA-dependent Golgi-localized factor for axon growth (Jang et al., 2004; Chin et al., 2006; Ruhl et al., 2019). *Lingo2* (encoding Leucine Rich Repeat and Ig domain Containing 2) with transcript distribution mainly to soma/axon upon RNAseq, as glycosylated transmembrane and extracellular protein. It has sequence homology to the NoGo receptor interactor LINGO1, which is induced upon nerve injury (Inoue et al., 2007), being responsible for axon regeneration and excitatory synapse organization. Its decrease was no longer significant at 28 dpl, possibly as a response to reinnervation.

Chronic downregulation with a minimum at 14 dpl during the regeneration stage concerned only *Lrrtm1* (encoding Leucine Rich Repeat Transmembrane Neuronal 1) with transcript distribution mainly to soma/axon upon RNAseq, as transmembrane protein that promotes axonal synaptogenesis mainly in interneurons, and being also crucial for adhesion together with neurexin/neuroligin interactions.

Chronic downregulation with a minimum at 28 dpl during the re-differentiation stage concerned two factors. On the one hand, *Ano3* (encoding Anoctamin 3, aka TMEM16C or DYT23 or DYT24) with glycosylated residues, eight transmembrane domains, and channel-like structure having calcium-dependent phospholipid scramblase activity, showing selective expression in neurons with transcript distribution to soma/axon, and interacting with Sortilin-1 coreceptor according to BioGRID database. On the other hand, *Chl1* (encoding Cell Adhesion Molecule with Homology to L1CAM, aka L1CAM2 or CALL), as ECM and cell adhesion factor implicated in axon development and synaptic plasticity, particularly in hippocampal interneurons, via binding to semaphorins, with both soluble and membranous forms promoting neurite outgrowth, in dependence on BACE1 cleavage. Overall, GCL chronic downregulations of axon/presynapse-distributed transcripts occurred only for transmembrane factors.

Chronic upregulations with maximal levels at 3 dpl during the repair stage were observed for two neurite modulators that have established main roles for axonogenesis: First and most importantly, *Smn1* (encoding Survival Motor Neuron Protein, aka Gemin 1) as structural component in the SMN complex responsible for the assembly of small nuclear ribonucleoproteins, which plays a selective critical role for the outgrowth, differentiation and survival of axons but possibly influences dendrite transport as well. Second, with a maximum at 3 dpl *Fgf5* as mostly neuron-expressed trophic ligand with important roles during neurogenesis.

Selective upregulation at 14-28 dpl with maximum at 28 dpl was documented for interneuron-expressed *Ptprn2* (encoding Protein Tyrosine Phosphatase Receptor Type N2, aka Phogrin or IA-2beta) as phosphatidylinositol phosphatase and PKA-dependent modulator of secretory vesicle dynamics with localization to synaptic boutons. This suggests that at this late stage a modulation of interneuron neuropeptide signals, secretome and extracellular matrix occurs. Taking into consideration that cellular upregulations in response to a lesion are probably compensatory efforts, such trophic regulators for axons/presynapses may represent crucial switches to promote repair and beneficial adjustments of hippocampal neuron projections.
